# Passivation of miniature microwave coplanar waveguides using a thin film fluoropolymer electret

**DOI:** 10.1038/s41598-021-03540-0

**Published:** 2021-12-16

**Authors:** Jaouad Marzouk, Vanessa Avramovic, David Guérin, Steve Arscott

**Affiliations:** University of Lille, CNRS, Centrale Lille, Univ. Polytechnique Hauts-de-France, UMR 8520-IEMN, 59000 Lille, France

**Keywords:** Engineering, Electrical and electronic engineering, Physics, Electronics, photonics and device physics

## Abstract

The insertion losses of miniature gold/silicon-on-insulator (SOI) coplanar waveguides (CPW) are rendered low, stable, and light insensitive when covered with a thin film (95 nm) fluoropolymer deposited by a trifluoromethane (CHF_3_) plasma. Microwave characterization (0–50 GHz) of the CPWs indicates that the fluoropolymer stabilizes a hydrogen-passivated silicon surface between the CPW tracks. The hydrophobic nature of the fluoropolymer acts as a humidity barrier, meaning that the underlying intertrack silicon surfaces do not re-oxidize over time—something that is known to increase losses. In addition, the fluoropolymer thin film also renders the CPW insertion losses insensitive to illumination with white light (2400 lx)—something potentially advantageous when using optical microscopy observations during microwave measurements. Capacitance–voltage (CV) measurements of gold/fluoropolymer/silicon metal–insulator-semiconductor (MIS) capacitors indicate that the fluoropolymer is an electret—storing positive charge. The experimental results suggest that the stored positive charge in the fluoropolymer electret and charge trapping influence surface-associated losses in CPW—MIS device modelling supports this. Finally, and on a practical note, the thin fluoropolymer film is easily pierced by commercial microwave probes and does not adhere to them—facilitating the repeatable and reproducible characterization of microwave electronic circuitry passivated by thin fluoropolymer.

## Introduction

Coplanar waveguides^1^ (CPW) are now common place in microwave electronic circuitry^2–4^. As is the case of most electrical and electronic components, miniaturization of CPW is proving beneficial in many applications areas, ranging from high-frequency telecommunications^5,6^, and emerging miniaturized tools^7^ for their automated characterization^8^, to quantum computing^9,10^ and biosensors^11^. In as much, both a physical understanding and a technological control of signal attenuation are major issues in future CPW engineering. For CPW patterned onto silicon, besides the well-understood loss mechanisms, i.e. conductor and substrate losses, it has been observed that intertrack surface-associated losses can have a contribution to signal attenuation. The influence of the depletion region in silicon and its impact on CPW losses was pointed out many years ago^12^. Since this, studies have been undertaken to understand such losses and their control has been attempted using techniques ranging from surface treatments and thin film deposition to etching intertrack material^13–25^. It was recently pointed out that surface-associated losses can be considerable for miniature CPW patterned onto silicon-on-insulator (SOI) wafers^26^. The selective removal of the native oxide from the silicon surface lying between miniature CPW tracks—to leave a hydrogen-terminated silicon surface—can significantly reduce the contribution of surface-associated losses in miniaturized CPW^26^. However, when the native oxide gradually grows back onto the unprotected hydrogen-terminated silicon surface between the metal tracks, the surface-associated losses increase. Although periodic removal of the native oxide resets the losses, this is not a viable long-term solution as the selective wet etch—hydrofluoric acid-based—is corrosive and can cause damage to chip materials. In an effort to try to solve this issue we turn here to thin film fluoropolymers, which have—in various forms—emerging applications in microwave engineering^27^. We investigate here the effect on the insertion losses of miniaturized CPW by depositing a thin film, hydrophobic fluoropolymer electret^28^ via plasma-enhanced chemical vapour deposition (PECVD) using a trifluoromethane (CHF_3_) plasma^29–31^ onto different CPW intertrack surfaces (hydrogen-terminated, native oxide, silicon dioxide) on suspended and non-suspended silicon structures. We do this to stabilize the surface-associated losses and render the CPW transmission characteristics insensitive to humidity and even illumination. To understand the losses in the CPW, gold/fluoropolymer/silicon metal–insulator-semiconductor (MIS) capacitors are fabricated and studied using capacitance–voltage (CV) measurements. The observations suggest the importance of the electret charging in the fluoropolymer and charge trapping at the fluoropolymer/silicon interface on the surface-associated losses in CPW.

## Results and discussions

### Characterization of coplanar waveguides

**Figure 1:**
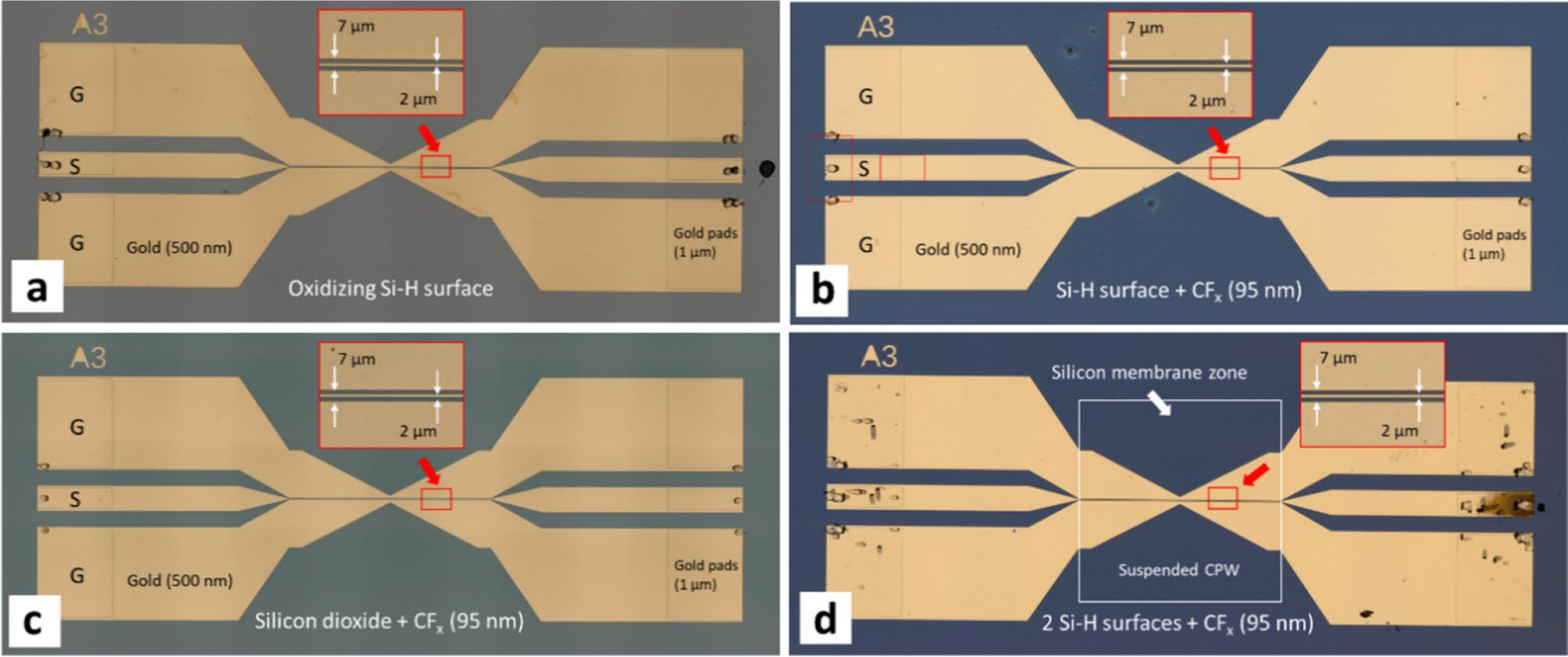
Miniaturized coplanar waveguides (CPW) microfabricated for the study using silicon-on-insulator (SOI) material. The insertion losses of the CPW were studied at GHz frequencies as a function of time, humidity, temperature, and illumination for different intertrack surfaces: (**a**) a hydrogen-terminated silicon surface exposed to air at room temperature, (**b**) a hydrogen-terminated silicon surface immediately covered with a thin film fluoropolymer (CF_x_), (**c**) a silicon dioxide thin film covered with a thin film fluoropolymer, and (**d**) a suspended membrane structure containing two hydrogen-terminated silicon surfaces covered with a thin film fluoropolymer. The CPW is composed of chromium/gold tracks patterned by lithography and evaporation. The 95 nm thick fluoropolymer layer was deposited using a trifluoromethane (CHF_3_) plasma. The hydrogen-terminated silicon was obtained by exposing the silicon surfaces to buffered hydrofluoric acid. The 100 nm thick silicon dioxide was deposited using chemical vapour deposition (CVD). In (**d**) the suspended silicon device layer is 20 µm thick. The insets indicate the miniaturized CPW portions which have a signal width of 2 µm and a ground-to-ground spacing of 7 µm. The white square in (**d**) indicates the suspended portion of the silicon device layer (800 × 800 µm)—obtained by silicon micromachining the handle wafer. These photographs were taken after testing to indicate the robustness of the miniature CPW to the various surface treatments. For the SOI, the device layer is 20 µm thick, the buried oxide is 1 µm thick, and the handle is 400 µm thick. The resistivity of the silicon in the SOI is > 1000 Ω cm. In each case, the miniature portion of the CPW is 800 µm long—and the large portion of the CPW has a signal width of 100 µm and a ground-to-ground spacing of 227 µm.

**Figure 2:**
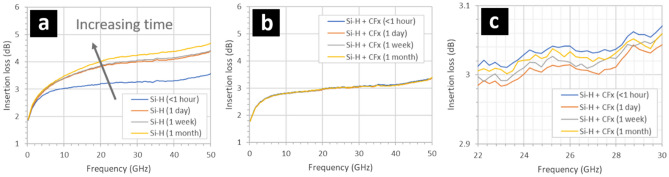
Insertion losses of gold/SOI coplanar waveguides having intertrack surfaces composed of (**a**) a hydrogen-terminated silicon surface (Si–H) and (**b**) a fluoropolymer-coated, hydrogen-terminated silicon surface (Si–H + CF_x_). (**c**) Insertion losses for a fluoropolymer-coated, hydrogen-terminated silicon intertrack CPW surface between 22 and 30 GHz. The hydrogen-terminated silicon surfaces were obtained by exposing the samples to buffered hydrofluoric acid. The 95 nm thick fluoropolymer was deposited using a trifluoromethane (CHF_3_) plasma. The native oxidation of the hydrogen-terminated silicon surfaces occurs at room temperature in air. The background light level was 350 lx.

Figure 1 shows the CPW samples fabricated for the study. The CPW are composed of chromium/gold (10/500 nm) tracks patterned onto commercial high-resistivity (HR) SOI wafers using lithographic and thin film deposition means—see Methods. The CPW are composed of a central signal track and two adjacent ground tracks. The samples have a central, 800 µm long, 50-Ω miniature CPW (signal width $S$ = 2 µm, ground-to-ground separation ($G$ − $G$) = 7 µm) imbedded between two larger, 50 Ω CPW (signal width $S$ = 100 µm, ground-to-ground separation ($G$ − $G$) = 227 µm) to enable contacting with commercial microwave probes. Two main types of samples have been fabricated: (1) CPW samples where the silicon handle wafer is present under the whole length of the metal tracks (Fig. 1a–c) and (2) CPW samples where the miniature 800 µm-long portion of the CPW is resting on a thin (20 µm thick) silicon membrane (Fig. 1d). Figure 1 also indicates another important aspect of the samples—the variability of the intertrack surface, i.e. the surface present between the metal tracks of the CPW. CPW having several types of intertrack surface were studied. These surfaces included: a bare hydrogen-terminated silicon surface undergoing water adsorption and native oxidation in air at room temperature (Fig. 1a), a hydrogen-terminated silicon surface that had been covered with a 95 nm-thick amorphous fluoropolymer (Fig. 1b) deposited by PECVD using trifluoromethane—see Methods. A 100 nm-thick silicon dioxide surface covered with the 95 nm-thick fluoropolymer (Fig. 1c), and a CPW sample involving two hydrogen-terminated silicon surfaces covered with the fluoropolymer (Fig. 1d). An intertrack surface composed of the fluoropolymer deposited onto a 1-month-old native oxide silicon surface was also studied (not shown in Fig. 1). The microwave characterization enabled the insertion losses (dB) of each CPW (type and surface condition) to be measured up to a frequency of 50 GHz—see Methods. Measurements were made under ambient (350 lx) and bright (2400 lx) white light. Some samples were dehydrated—via annealing at a temperature of 180 °C—prior to measurements; some samples were exposed to humidity—via immersion in deionized water—prior to measurements.

Figure 2 shows the evolution of the insertion losses over time (1 month) of two types of CPW having two different intertrack surfaces: a hydrogen-terminated silicon surface (Fig. 2a) and a fluoropolymer coated, hydrogen-terminated silicon surface (Fig. 2b). Figure 2a shows the variation of insertion losses of a CPW having an intertrack surface composed of bare, hydrogen-terminated silicon exposed to air at room temperature. Following exposure of the intertrack silicon surface to buffered hydrofluoric acid, the CPW were measured at four times: < 1 h, 1 day, 1 week, and 1 month. The insertion losses gradually increase over 1 month, e.g. from 3.5 to 4.6 dB at 50 GHz—the largest change being at higher frequency. The gradual increase in the insertion losses over a period of 1 month can be attributed solely to the increase in surface-associated losses in the miniature portion of the CPW—thought to be due to free carrier accumulation at the intertrack silicon surface^26^. When exposed to air and room temperature over a period of 1 month, the a hydrogen-terminated silicon surface^32–34^ between the tracks will adsorb water and form what is known as a thin ‘native’ oxide^35–37^. These effects are known to influence the surface conductivity of the silicon due to the presence or not of carriers at the silicon surface^38–40^.

**Figure 3:**
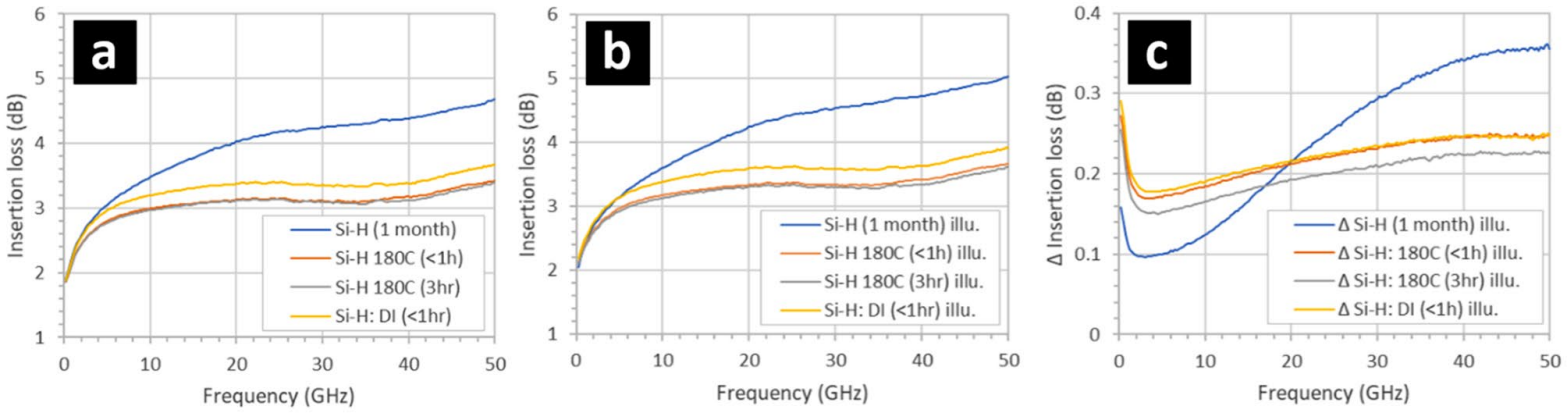
The effect of illumination, temperature, and humidity on the insertion losses of gold/SOI coplanar waveguides having 1-month old oxidized hydrogen-terminated (Si–H) silicon surfaces situated between the metal tracks. The insertion losses of the CPW under (**a**) ambient lighting and under (**b**) white light illumination, (**c**) the increase in the insertion losses in the CPW when illuminated with white light. The hydrogen-terminated silicon surfaces were obtained by exposing the samples to buffered hydrofluoric acid. The native oxidation of the silicon surfaces occurred at room temperature in air over a period of > 1 month. The illumination was white light at 2400 lx—see Methods. The samples were heated to 180 °C for 3 min in air. The samples were exposed to deionized wafer (resistivity > 18 Mohms cm) for 3 min.

Figure 2b shows the variation of insertion losses with time of a CPW having an intertrack surface composed of bare, hydrogen-terminated silicon that has been rapidly covered with a 95 nm-thick thin film of fluoropolymer using PECVD—see Methods. It can be observed that the effect of the fluoropolymer is to slightly reduce the insertion losses compared to the newly-formed hydrogen-terminated silicon surface—see Fig. 3a (cf. blue line in Fig. 2). In addition, the insertion losses do not increase over a period of 1 month—in stark contrast to the case of the bare hydrogen-terminated silicon surface (Fig. 1a). Indeed, the insertion losses remain very stable with time, e.g. ± 0.04 dB over a period of 1 month—see Fig. 2c. The results indicate that the effect of the fluoropolymer is to ‘stabilize’ the hydrogen-terminated silicon surface. The hydrophobic nature of the fluoropolymer is likely to be acting as a humidity barrier—it is known that humidity adsorption plays a key role in the native oxidation of a silicon surface^41^.

Figure 3 shows the effect of illumination with white light, temperature, and humidity on the insertion loss of CPW having 1-month old oxidized, hydrogen-terminated silicon surfaces situated between the metal tracks. Firstly, exposure of the sample to heat (180 °C) leads to a reduction of the insertion losses—see Fig. 3a. The temperature cycle results in insertion losses very close to those recorded for a hydrogen-terminated silicon intertrack surface—cf. Figure 2a. The losses do not evolve over a 3-h period following the heat treatment. Following this, exposure of the CPW sample to deionized water leads only to a small increase in the insertion losses—but not to the level observed for the 1-month old oxidized hydrogen-terminated silicon intertrack surfaces. The effect of illumination with bright white light is to cause the insertion losses to increase considerably in all samples—see Fig. 3b. The differences in the losses due to illumination are plotted in Fig. 3c.

**Figure 4:**
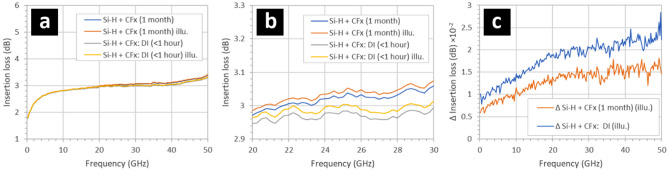
The effect of illumination and humidity on the insertion losses of 1-month old gold/SOI coplanar waveguides having fluoropolymer (CF_x_) coated hydrogen-terminated (Si–H) silicon surfaces situated between the metal tracks. The insertion losses of the CPW under (**a**) ambient lighting and white light illumination, (**b**) the insertion losses plotted between 20 and 30 GHz, and (**c**) the increase in the insertion losses in the CPW when illuminated with white light. The hydrogen-terminated silicon surfaces were obtained by exposing the samples to buffered hydrofluoric acid—see Methods. The 95 nm thick fluoropolymer was deposited using a CHF_3_ plasma—see Methods. The samples had been exposed to air for > 1 month. The illumination was white light at 2400 lx—see Methods. The samples were exposed to deionized wafer (resistivity > 18 Mohms cm) for 3 min.

Figure 4 shows the effect of illumination with white light and humidity on 1-month old CPW samples which have hydrogen-terminated surfaces coated with fluoropolymer. It was observed that relatively bright white light (2400 lx) and humidity (3 min in deionized water) had little effect upon the insertion losses—see Fig. 4a. Indeed, emersion of the sample into deionized water actually led to a small decrease of the insertion losses—see Fig. 4b. In addition, the CPW sample was now highly insensitive to illumination, 2.5 × 10^–2^ dB—see Fig. 4c and cf. Figure 3c. Note that the transmission of thin film fluoropolymer to white light is high (> 95%)^42^, meaning photogeneration of free carriers in the CPW intertrack silicon surface must occur—although they are not contributing to microwave losses.

**Figure 5:**
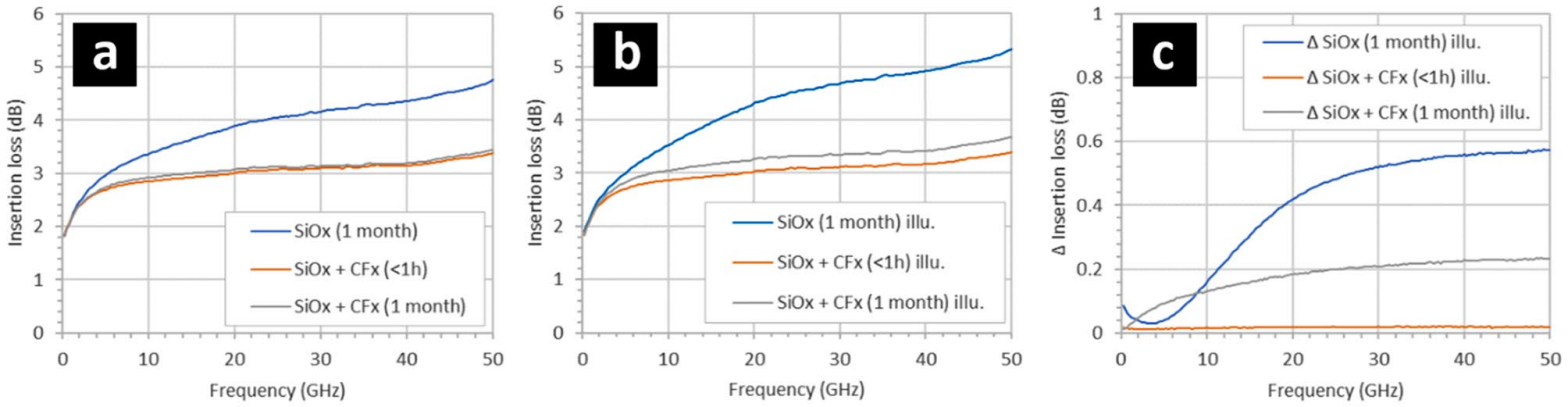
The effect on the insertion losses of gold/SOI coplanar waveguides by depositing a fluoropolymer (CF_x_) directly onto the 1-month-old native oxidized silicon surfaces situated between the metal tracks. The insertion losses of the CPW recorded under (**a**) ambient lighting and under (**b**) white light illumination, (**c**) the increase in the insertion losses in the CPW when illuminated with white light. The hydrogen-terminated silicon surfaces were obtained by exposing the samples to buffered hydrofluoric acid—see Methods. The native oxidation of the silicon surfaces occurred at room temperature in air over a period of > 1 month. The 95 nm thick fluoropolymer was deposited using a CHF_3_ plasma—see Methods. The illumination was white light at 2400 lx. The background light level (non-illuminated) was 350 lx.

**Figure 6:**
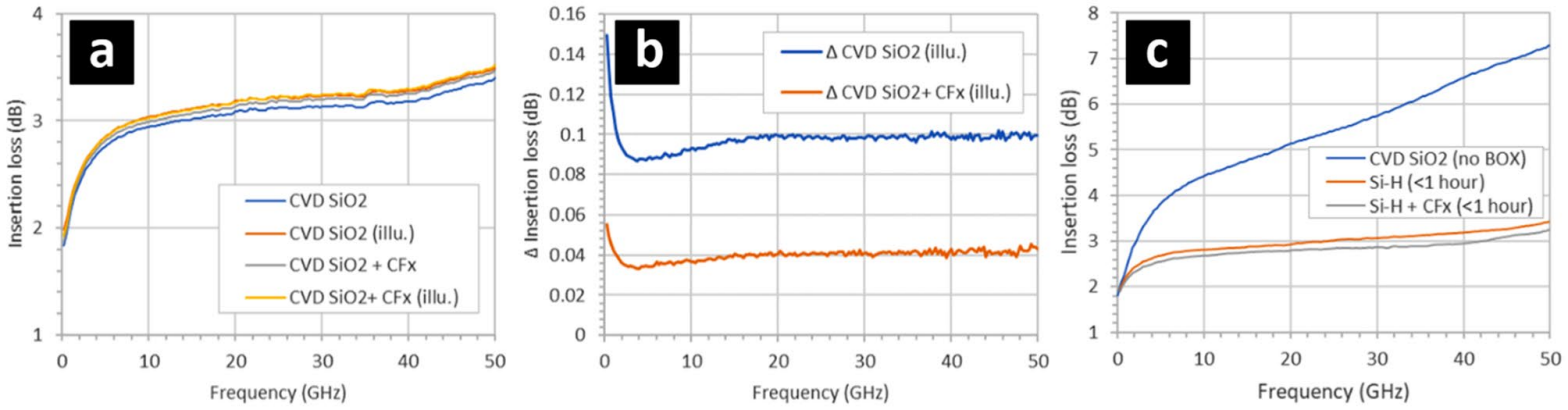
(**a**) The effect on the insertion losses of coplanar waveguides by depositing a fluoropolymer (CF_x_) directly onto the oxidized silicon surfaces situated between the metal tracks. (**b**) The relative change in the losses shown in (**a**). (**c**) The effect of on the losses of removing the CVD oxide in suspended miniature CPW and depositing the fluoropolymer on both sides of the silicon membrane. The oxidation of the silicon (100 nm) surfaces was obtained using chemical vapour deposition (CVD)—see Methods. The 95 nm thick fluoropolymer was deposited using an CHF_3_ plasma—see Methods. The illumination was white light at 2400 lx. The background light level (non-illuminated) was 350 lx.

Figure 5 shows the effect on the insertion losses of CPW by depositing a thin film fluoropolymer directly onto a 1-month old native oxidized silicon surface situated between the CPW metal tracks. The native oxide formed on a hydrogen-terminated silicon surface in air and at room temperature for 1 month—see Methods. The addition of fluoropolymer thin film onto the CPW intertrack surfaces reduces the insertion losses considerably—see Fig. 5a. The insertion losses fall to a level comparable with those observed for a freshly-treated hydrogen-terminated silicon surface that has been immediately deposited with fluoropolymer—cf. Figure 2b. In addition, the insertion losses are stable in time; after 1 month the losses remain the same as those recoded after < 1 h—see Fig. 5a. However, in this case the insertion losses now increase with illumination with white light (2400 lx)—see Fig. 6b. Interestingly, the insertion losses are more sensitive to illumination than CPW samples coated with fluoropolymer directly after treatment with buffered HF—see Fig. 5c, and cf. Figure 4c. Note that exposure of a thin native silicon oxide to the trifluoromethane plasma will probably result in its removal in the very early phase of the fluoropolymer deposition. The results suggest that the resulting interface between the oxidized silicon and the fluoropolymer following exposure to the CHF_3_ plasma (used both to deposit fluoropolymers^29^ and also etch silicon oxide^43^) is similar to the hydrogen-terminated silicon and the fluoropolymer interface following exposure to the plasma—cf. Figure 4. However, the behaviour of the losses under illumination of these two intertrack surfaces is not the same.

blue.

Figure 6 shows two technologically-important results. Figure 6a shows the effect on the insertion losses when the fluoropolymer is deposited onto an CPW intertrack surface composed of a silicon dioxide thin film on top of an HR SOI wafer. The silicon dioxide film is 100 nm thick and deposited using CVD—see Methods. Interestingly, the insertion losses are slightly increased when the fluoropolymer is present—see Fig. 6a. However, the insertion losses become considerably less sensitive to illumination in the presence of the 95 nm-thick fluoropolymer on the surface of the silicon dioxide—see Fig. 6b. With the thin film silicon dioxide present, the insertion losses are larger than with the fluoropolymer deposited directly onto the silicon surface (hydrogen-terminated or native oxide) cf. Figure 2 and Fig. 5. The necessity of certain technological processes means that removal of the thin silicon dioxide between the CPW tracks is sometimes unavoidable. In this context, Fig. 6c shows the effect on the insertion losses by removing the CVD silicon dioxide between the CPW tracks on a suspended silicon membrane (Fig. 1d) and subsequently depositing the fluoropolymer onto both sides of the silicon device layer membrane. Note that in this structure, the BOX was not present due to the nature of the technological process—a condition which we have already observed to cause high losses in suspended miniature CPW^26^. The results indicate that we now have a technological solution to this problem using the thin film fluoropolymer. Finally, for all microwave measurements the measured return losses (S_11_ and S_22_) were less than 15 dB due to all CPW (large and small gap) being ~ 50 ohms. In addition, it was verified that S_21_ = S_12_ to within measurement error. This indicates good CPW matching, meaning that the measured insertion loss differences between samples is due to surface treatments and film depositions. The repeatability of the microwave measurements is excellent from measurement to measurement for a given CPW and also from CPW to CPW, typically ± 0.05 dB. The reason for this is severalfold: the CPW dimensions (lateral and thickness) are very accurately defined by electron beam lithography and thermal evaporation, the microwave probe contacting is very reproducible, and the microwave measurement tool (VNA) is very accurate and stable.

To gain an understanding of how the fluoropolymer is influencing the surface-associated part of the microwave insertion losses we can look at the electrical behaviour of metal–insulator-semiconductor (MIS) and metal–insulator-metal (MIM) capacitors using the fluoropolymer as the insulator. This will be the subject of the next section.

### Electrical characterization of MIM and MIS capacitors

**Figure 7:**
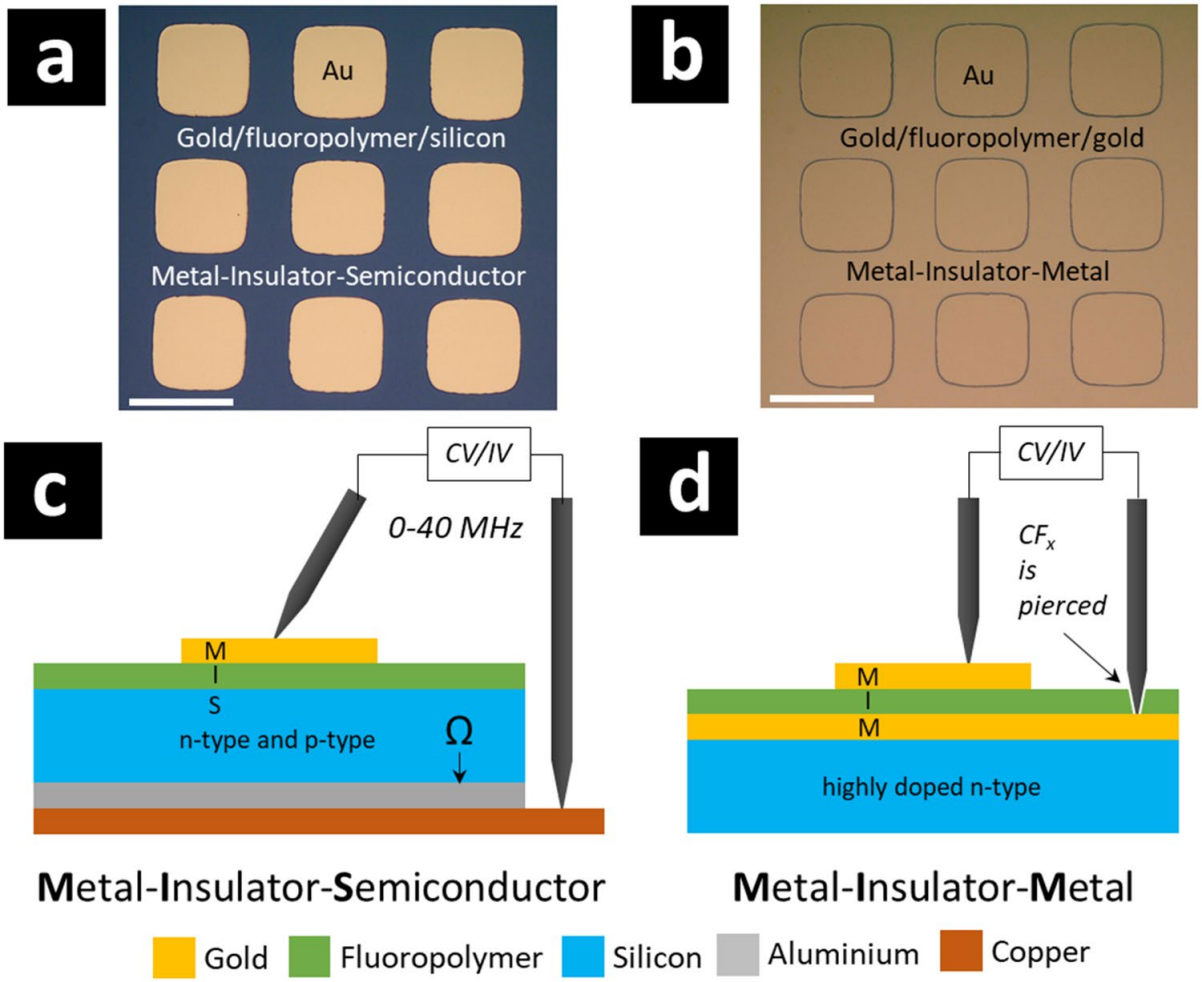
Metal–insulator-semiconductor (MIS) and metal–insulator-metal (MIM) structures microfabricated for the study. (**a**) The MIS structures are composed of gold (200 nm)/fluoropolymer (95 nm) thin films deposited onto p-type and n-type silicon wafers. (**b**) The MIM structures are composed of gold (200 nm)/fluoropolymer (95 nm)/gold (200 nm) thin films deposited onto n-type silicon wafers. Depending on the silicon doping type, the rear face of the MIS structure incorporates an ohmic contact obtained by ion implantation/annealing of boron (p^+^) or phosphorous (n^+^) covered by a thin film of evaporated, annealed aluminium. For the current–voltage and the capacitance–voltage characterization, the measurement set-up for the MIS and the MIM devices are shown in (**c**) and (**d**). The gold top contact dots shown in (**a**) and (**b**) are obtained by evaporation via a physical shadow mask; the gold dots have a surface area of approximately 7 × 10^–4^ cm^2^. In the case of the characterisation of the MIM structures (IV and CV), the soft fluoropolymer thin film is easily pierced by the sharp measurement probe to provide an electrical contact to the bottom gold layer. In contrast, the characterization of both MIM and MIS structures is facilitated using a ‘blunt’ needle probe for the top contact. The scale bars on (**a**) and (**b**) are 300 µm.

Figure 7 shows the gold/fluoropolymer/silicon metal–insulator-semiconductor (MIS) and the gold/fluoropolymer/gold metal–insulator-metal (MIM) capacitors fabricated for the study; and how they were probed for the current–voltage (IV) and capacitance–voltage (CV) characterization—see Methods.

**Figure 8:**
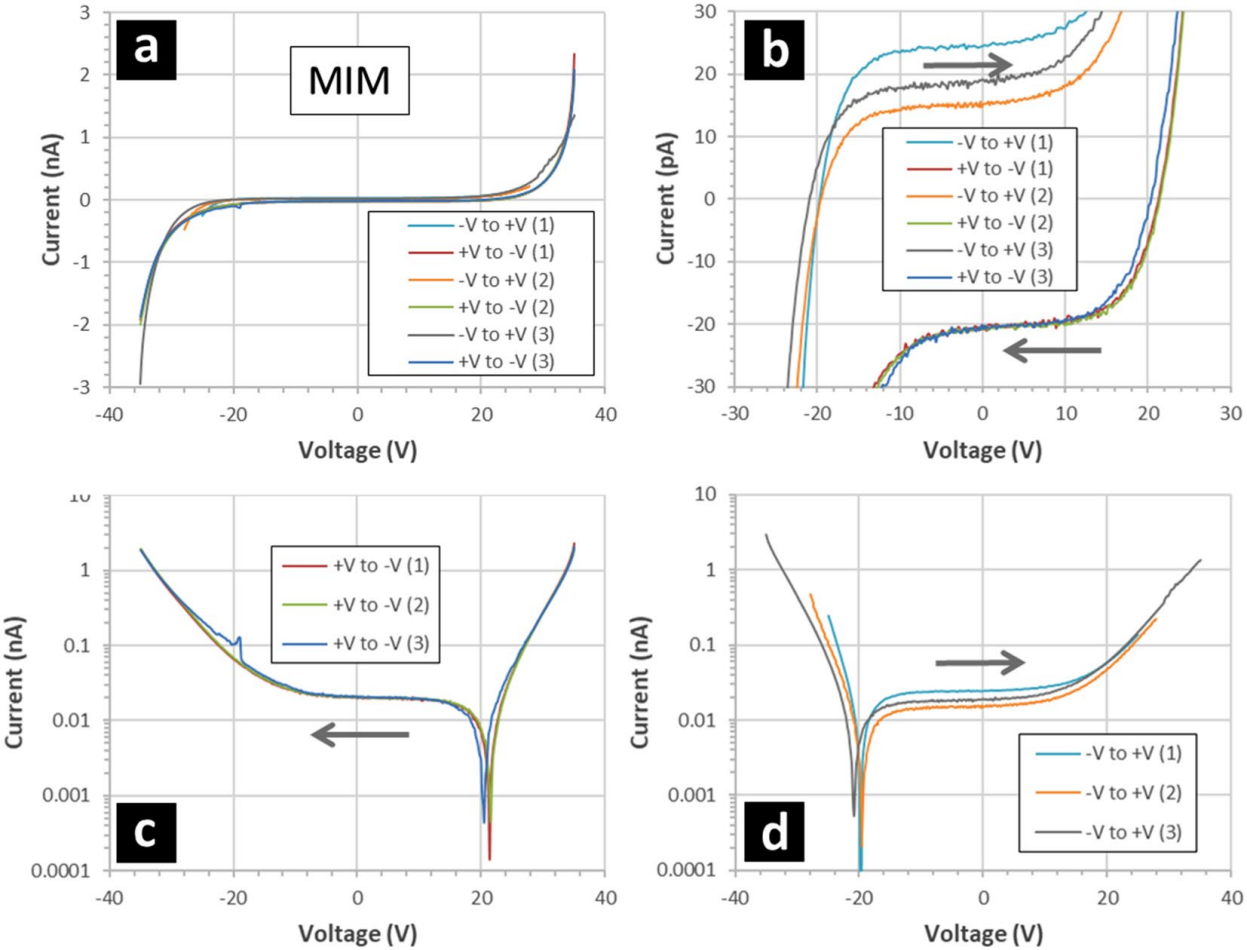
Current–voltage measurements of MIM capacitors. The MIM capacitors are composed of gold/fluoropolymer/gold thin films deposited onto silicon wafers. The gold dots are 200 nm thick and have an area of 7 × 10^–4^ cm^2^. The fluoropolymer is 95 nm thick and deposited using a CHF_3_ plasma. The starting polarity of the applied voltage sweep is alternated. The grey arrows indicate the voltage sweep direction: varied between ‘ + V to − V’ and ‘− V to + V’ sequentially. (**a**) Indicates the full voltage sweeps, (**b**) shows a zoom from − 30 to + 30 V, (**c**) shows + V to − V voltage sweeps with current plotted logarithmically, and (**d**) shows − V to + V voltage sweeps with current plotted logarithmically.

Figure 8 shows the results of current–voltage measurements performed on the MIM capacitors. First, the IV characteristics appear to be symmetrical in voltage and with the direction of the voltage sweep—see Fig. 8a. However, on closer inspection and if one looks at small currents at lower voltages (Fig. 8b), then a reproducible hysteresis is apparent in the results. This hysteresis depends on the voltage sweep direction—the zero current crossing point is shifted either to the left or the right (~ ± 20 V) depending on the voltage sweep direction. In addition, by plotting the current on a logarithmic scale, an asymmetry is apparent in how the current evolves with the voltage—see Fig. 8c and Fig. 8d. Note that the absolute values of the current are plotted in Fig. 8c and Fig. 8d—i.e. the polarity of the current changes once the zero-current point is passed, cf. Figure 8b. The IV measurements suggest a breakdown field of the order of 3–4 × 10^8^ Vm^-1^; this is high but the breakdown strength of fluoropolymer thin films in known to be large^44^.

**Figure 9:**
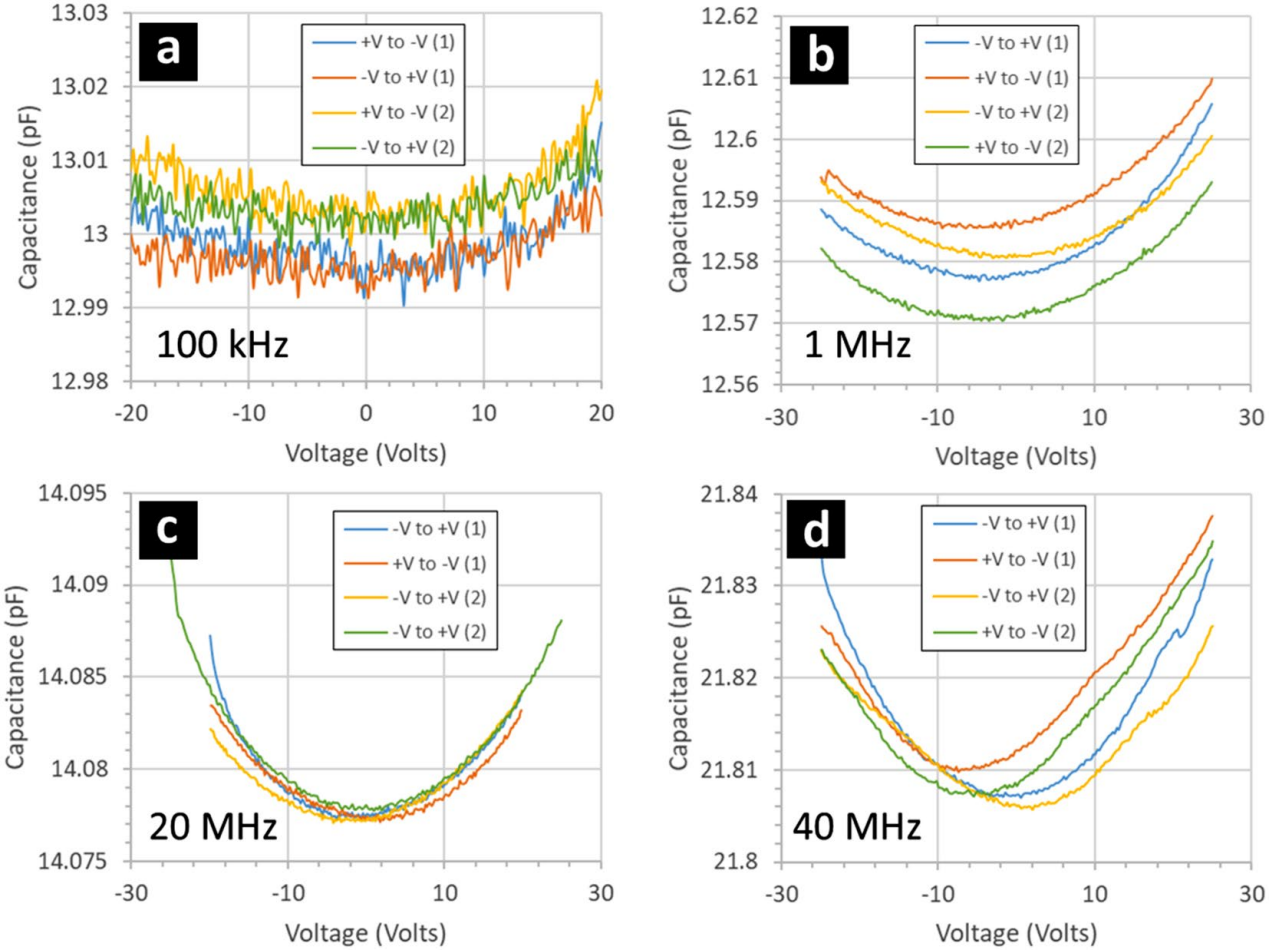
Capacitance–voltage measurements of gold/fluoropolymer/gold MIM capacitors when the direction of the applied voltage sweep is alternated. The MIM capacitors are composed of gold/fluoropolymer/gold thin films deposited onto highly-doped silicon wafers. The gold dots are 200 nm thick and have an area of 7 × 10^–4^ cm^2^. The fluoropolymer (CF_x_) is 95 nn thick and deposited using a CHF_3_ plasma. The small-signal measurement frequency is (**a**) 100 kHz, (**b**) 1 MHz, (**c**) 20 MHz, and (**d**) 40 MHz.

Figure 9 shows the results of capacitance–voltage measurements performed on the gold/fluoropolymer/gold MIM capacitors. First, the measurements enable an extraction of the dielectric constant of the fluoropolymer to be 2.04 ± 0.05 up to 20 MHz. This is in good agreement with values in the literature^42^. The dielectric constant of fluoropolymer deposited using PECVD of CHF_3_—using comparable deposition conditions to those used here—was measured to be 2.2^31^. This is very close to the value measured using bulk material ~ 2.1 up to GHz frequencies, even THz frequencies^45^. Second, the measurements indicate a weak, but apparent, variation of the capacitance with the applied voltage. The symmetrical quadratic change of the capacitance with voltage polarity can be explained by electrostriction of the fluoropolymer^46,47^. As with the IV measurements, the CV measurements indicate a weak capacitance hysteresis as a function of voltage sweep direction—e.g. Figure 9d.

**Figure 10:**
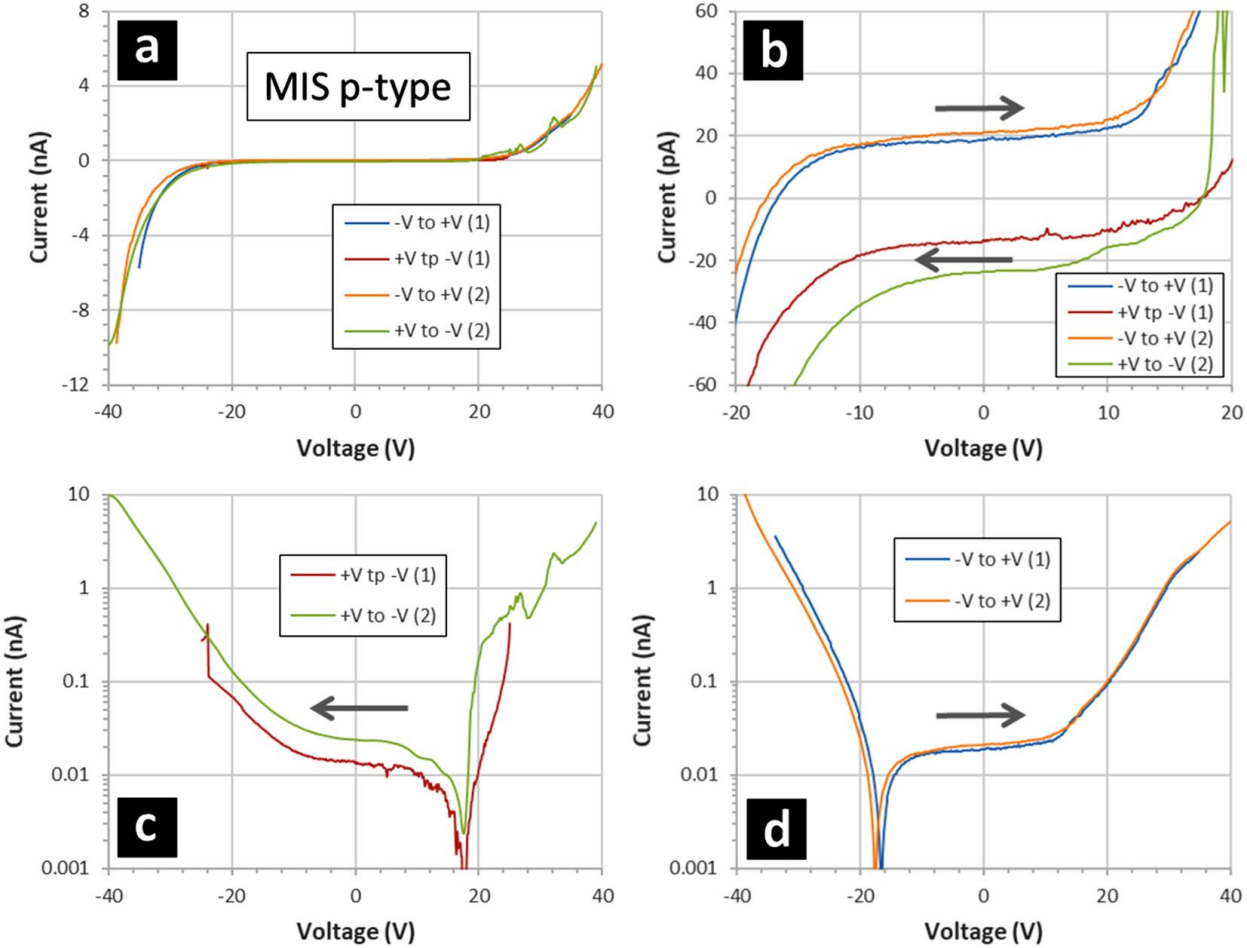
Current–voltage measurements of p-type MIS capacitors. The MIS capacitors are composed of gold/fluoropolymer thin films deposited onto p-type silicon wafers—see Methods. The p-type resistivity is 0.1–0.5 Ω cm. The gold dots are 200 nm thick and have an area of 7 × 10^–4^ cm^2^. The fluoropolymer is 95 nm thick and deposited using a CHF_3_ plasma. The starting polarity of the applied voltage sweep is alternated. The grey arrows indicate the voltage sweep direction: varied between ‘+ V to − V’ and ‘− V to + V’ sequentially. (**a**) Indicates the full voltage sweeps, (**b**) shows a zoom from − 20 V to + 20 V, (**c**) shows + V to − V voltage sweeps with current plotted logarithmically, and (**d**) shows − V to + V voltage sweeps with current plotted logarithmically.

**Figure 11:**
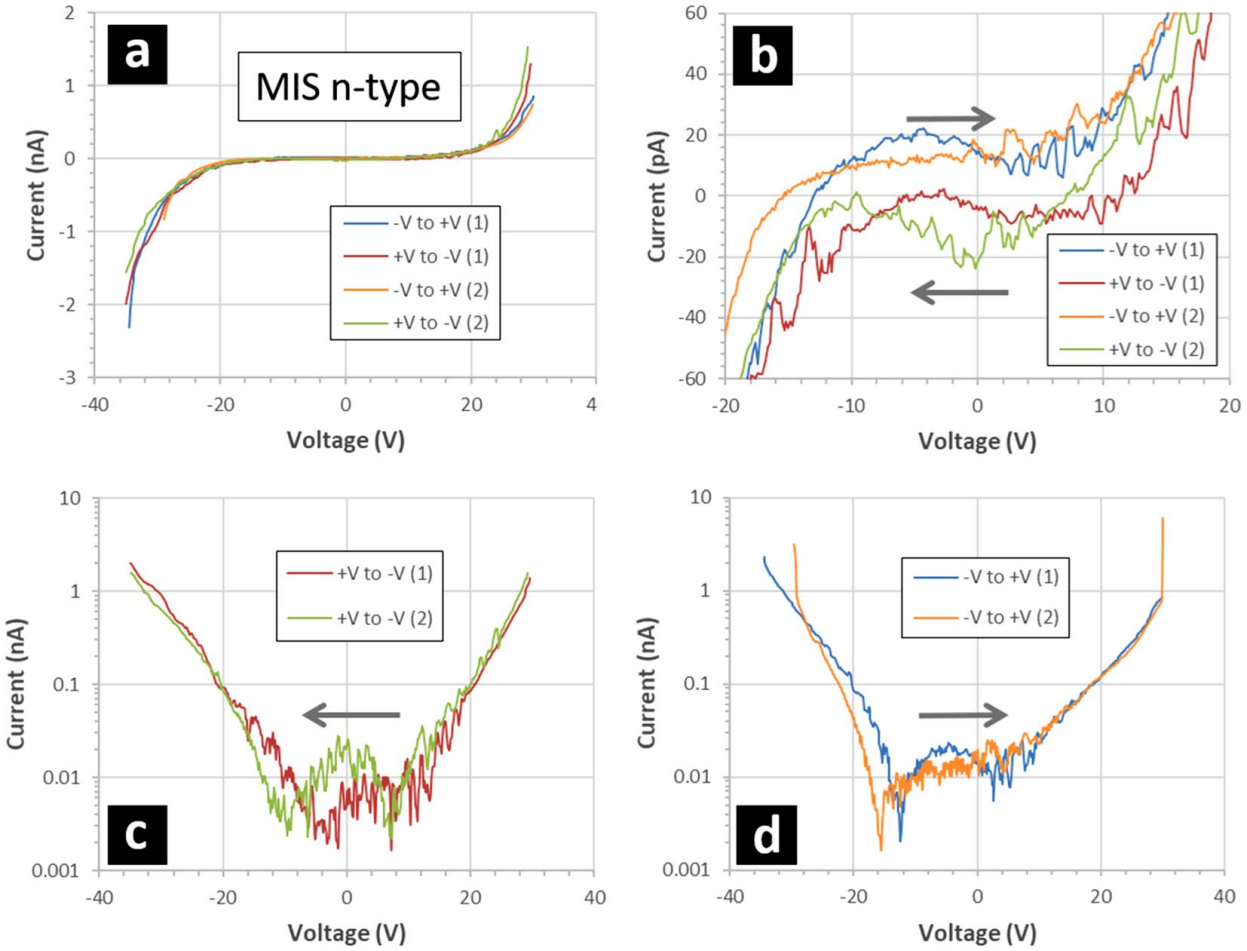
Current–voltage measurements of n-type MIS capacitors. The MIS capacitors are composed of gold/fluoropolymer thin films deposited onto n-type silicon wafers—see Methods. The n-type resistivity is 0.03–0.05 Ω cm. The gold dots are 200 nm thick and have an area of 7 × 10^–4^ cm^2^. The fluoropolymer is 95 nm thick and deposited using a CHF_3_ plasma. The starting polarity of the applied voltage sweep is alternated. The grey arrows indicate the voltage sweep direction: varied between ‘+ V to − V’ and ‘− V to + V’ sequentially. (**a**) Indicates the full voltage sweeps, (**b**) shows a zoom from − 20 V to + 20 V, (**c**) shows + V to − V voltage sweeps with current plotted logarithmically, and (**d**) shows − V to + V voltage sweeps with current plotted logarithmically.

Figures 10 and 11 show the results of current–voltage measurements performed on the MIS capacitors when the silicon doping is p-type (Fig. 10) and n-type (Fig. 11). As is the case with the IV characteristics of the MIM capacitors, the MIS capacitors indicate a hysteresis in the current which depends on the direction of the voltage sweep—see Figs. 10b and 11b. Again, the zero current crossing point is shifted to the left or the right depending on the voltage sweep direction, e.g. at − 18 V for p-type silicon when voltage sweep direction is from − V to + V—see Fig. 10d. At lower voltages, the values of the current is ~  ± 20 pA depending on the voltage sweep direction—similar to the IV characteristics of the MIM capacitors. The open circuit current of the measurement set-up was evaluated to be < 10 pA and independent of the voltage sweep direction. Also, the doping type has apparently no effect on the position of the zero current crossing point—the results being similar to those of the MIM capacitors. This indicates that the hysteresis effect is not associated with the underlying silicon—but rather the nature of the fluoropolymer thin film.

**Figure 12:**
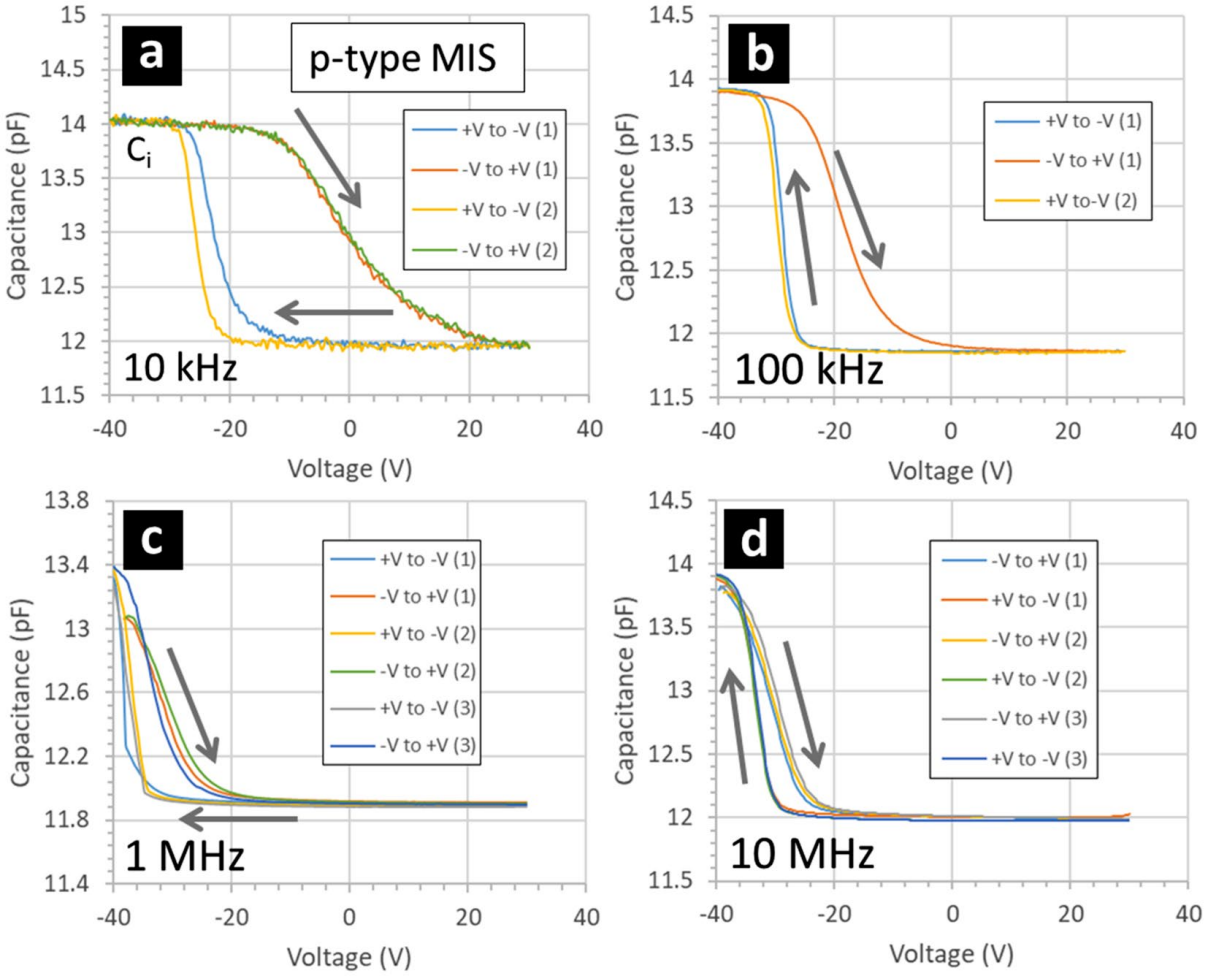
Capacitance–voltage measurements of gold/fluoropolymer/p-type silicon MIS capacitors when direction of the applied voltage sweep is alternated. The MIS capacitors are composed of gold/fluoropolymer thin films deposited onto p-type silicon wafers—see Methods. The p-type resistivity is 0.1–0.5 Ω cm. The gold dots are 200 nm thick and have an area of 7 × 10^–4^ cm^2^. The fluoropolymer (CF_x_) is 95 nm thick and deposited using a CHF_3_ plasma. The grey arrows indicate the voltage sweep direction: varied between ‘+ V to − V’ and ‘− V to + V’ sequentially. The small-signal measurement frequency is (**a**) 10 kHz, (**b**) 100 kHz, (**c**) 1 MHz, and (**d**) 10 MHz.

**Figure 13:**
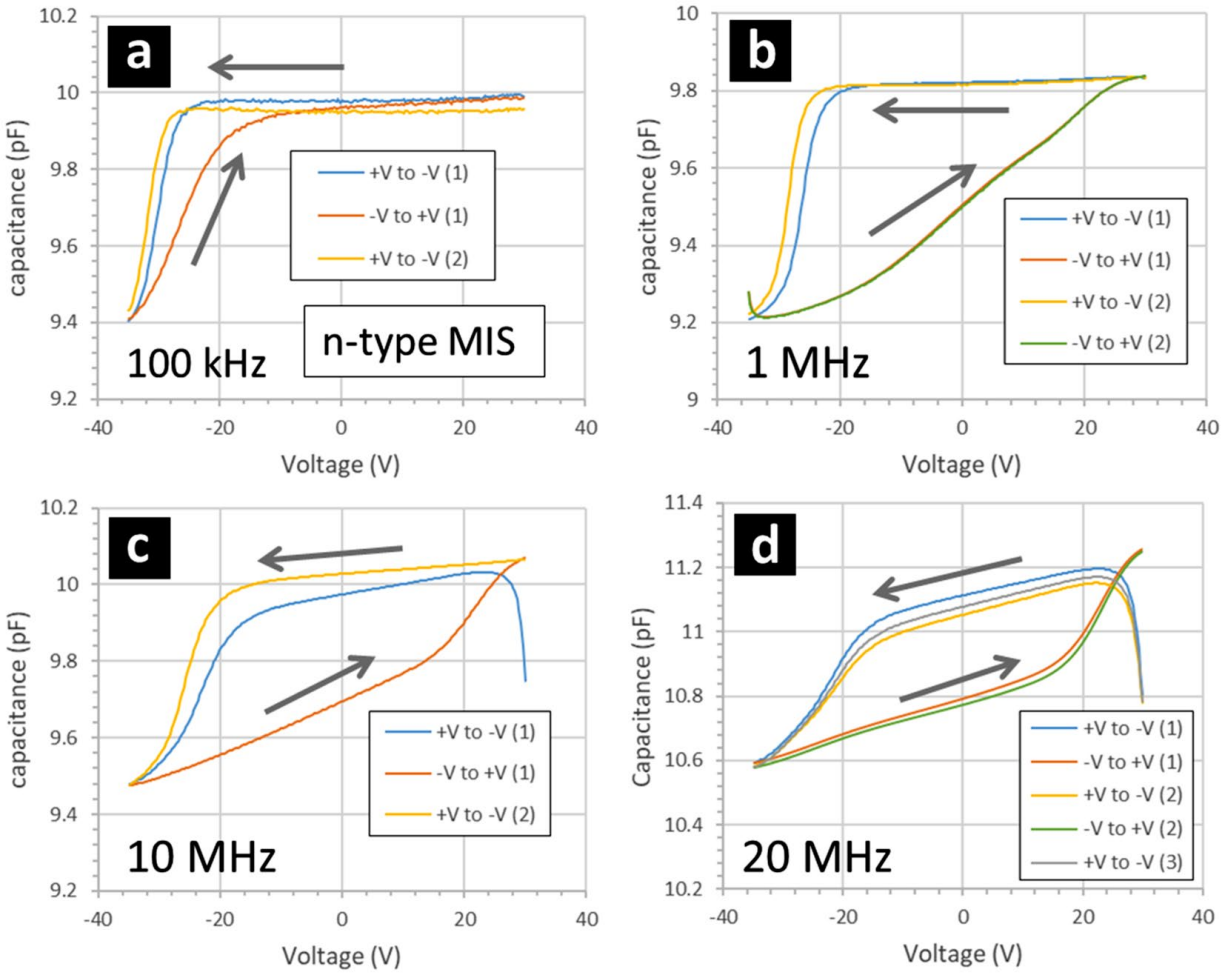
Capacitance–voltage measurements of gold/fluoropolymer/n-type silicon MIS capacitors when direction of the applied voltage sweep is alternated. The MIS capacitors are composed of gold/fluoropolymer thin films deposited onto n-type silicon wafers—see Methods. The n-type resistivity is 0.03–0.05 Ω cm. The gold dots are 200 nm thick and have an area of 7 × 10^–4^ cm^2^. The fluoropolymer (CF_x_) is 95 nm thick and deposited using a CHF_3_ plasma. The grey arrows indicate the voltage sweep direction: varied between ‘+ V to − V’ and ‘-V to + V’ sequentially. The small-signal measurement frequency is (**a**) 100 kHz, (**b**) 1 MHz, (**c**) 10 MHz, and (**d**) 20 MHz.

Figures 12 and 13 show capacitance–voltage curves obtained by measuring the gold/fluoropolymer/silicon MIS capacitors. Results are shown for p-type (Fig. 12) and n-type (Fig. 13) silicon at different measurement frequencies. The capacitance–voltage measurements reveal a number of interesting observations. First, the CV curves are all shifted towards negative voltage bias—for both p-type (Fig. 12) and n-type silicon (Fig. 13). This shift is more apparent when the sweep voltage starts at a positive voltage value. Second, the CV curves all display hysteresis with respect to the direction of the voltage sweep—for both p-type (Fig. 12) and n-type silicon (Fig. 13). In the case of p-type silicon, the hysteresis is clockwise whereas in the case of n-type silicon, the hysteresis is anticlockwise. Note that a similar CV curve is traced if several voltage sweeps (starting with the same voltage polarity—negative or positive) are used to sequentially bias the MIS capacitor. Note also that no hysteresis looping of the CV curves was found when measuring the MIM capacitors, cf. Figure 9. Finally, the shape of the CV curves is a modified by the small-signal measurement frequency. In the case of the p-type silicon (Fig. 12), the hysteresis is reduced as the measurement frequency increases—with the voltage shift being maintained. In the case of n-type silicon (Fig. 13), the hysteresis is somewhat maintained at high frequency—but the CV curve is flattened out.

Let us first consider the voltage shifts in the CV curves. A voltage shift of the CV curve to the left (towards negative voltage) for both a p-type and an n-type semiconductor is indicative of the presence of positive charges in the insulator^48^. The experimental value of the voltage shift (+ V to − V) is of the order of − 25 V to − 30 V for both p-type and n-type MIS capacitors. The CV measurements of the MIS capacitors therefore suggest that the fluoropolymer is an electret. Note that the shifted CV curves appear as what one would expect^49,50^ on the + V to − V voltage sweep—this is true for both p-type (Fig. 12) and n-type (Fig. 13) silicon. This in contrast to the − V to + V voltage sweep capacitance which display stretching out and hysteresis^49,50^ and which will be discussed after. These observations are consistent with the PECVD parameters for fluoropolymer deposition—the negative voltage bias on the sample during the deposition of the fluoropolymer film results in positive ions in the plasma being attracted towards, and incorporated into, the growing fluoropolymer film^51^.

Let us now consider the apparent hysteresis in the CV measurements of the MIS capacitors—obtained when changing the voltage sweep direction. For voltage sweeps which start with a negative voltage, the shift in the CV curve is lower compared to the shift in the CV curve when starting the voltage sweeps from a positive value—this is what causes the CV curves to display what are commonly known as CV ‘spreading out’ and ‘hysteresis loops’. First, hysteresis loops are well known in MIS junctions—ranging from the classic MOS^50,52,53^ to MIS structures involving polymers^54–60^. Indeed, the subject remains scientifically and technologically important for semiconductor devices^61–67^. When used as gate materials, the voltage-switchable dipoles in fluoropolymers are thought to lead to hysteresis in the electronic properties of devices^68,69^. The bias-dependent shifting of the CV curve, which causes the hysteresis, is attributed to mobile charges in the insulator. An anticlockwise/clockwise hysteresis loop in a CV curve of MIS capacitor indicates a positive/negative carrier injection into the semiconductor with subsequent trapping^49,50^. In addition, the CV results indicate the presence of interface trapped charges. For the p-type MIS, this manifests itself as a stretching out of the CV curve in the − V to + V voltage sweeps at lower frequency^49,50^. However, the n-type MIS do not indicate this. As the measurement frequency increases for the p-type MIS, the hysteresis reduces but the shift of the CV curve remains. This indicates that the charge effect remains at high frequency but that trapping at the silicon/fluoropolymer interface or movement of charge in the polymer are not important at higher frequencies. This suggests that the lower stable losses in the CPW and the insensitivity to illumination are not related to carriers trapping at the interface. The losses are governed by depletion of holes by the positive charges in the fluoropolymer and trapping of electrons in the fluoropolymer attracted by the positive fixed charge. Note that in the current work we refrain from extracting the trap density from the CV measurements as even in near-ideal CV measurements extracted values can be prone to errors^50^. However, the doping of the silicon wafers was estimated from the CV profiles. This is done by plotting the inverse squared capacitance ($1/C^{2}$) as a function of voltage $V$ and using a suitable coefficient^50^. In the carrier depletion part of the CV curves, the $1/C^{2}$ vs $V$ plots are linear. For the p-type silicon wafer, the doping was evaluated to be 6.7 × 10^16^ cm^−3^ for the + V to − V voltage sweeps. For the n-type silicon wafer, the doping was evaluated to be 1.3 × 10^17^ cm^−3^ for the + V to − V voltage sweeps.

### Characterization of the fluoropolymer using FTIR spectroscopy

**Figure 14:**
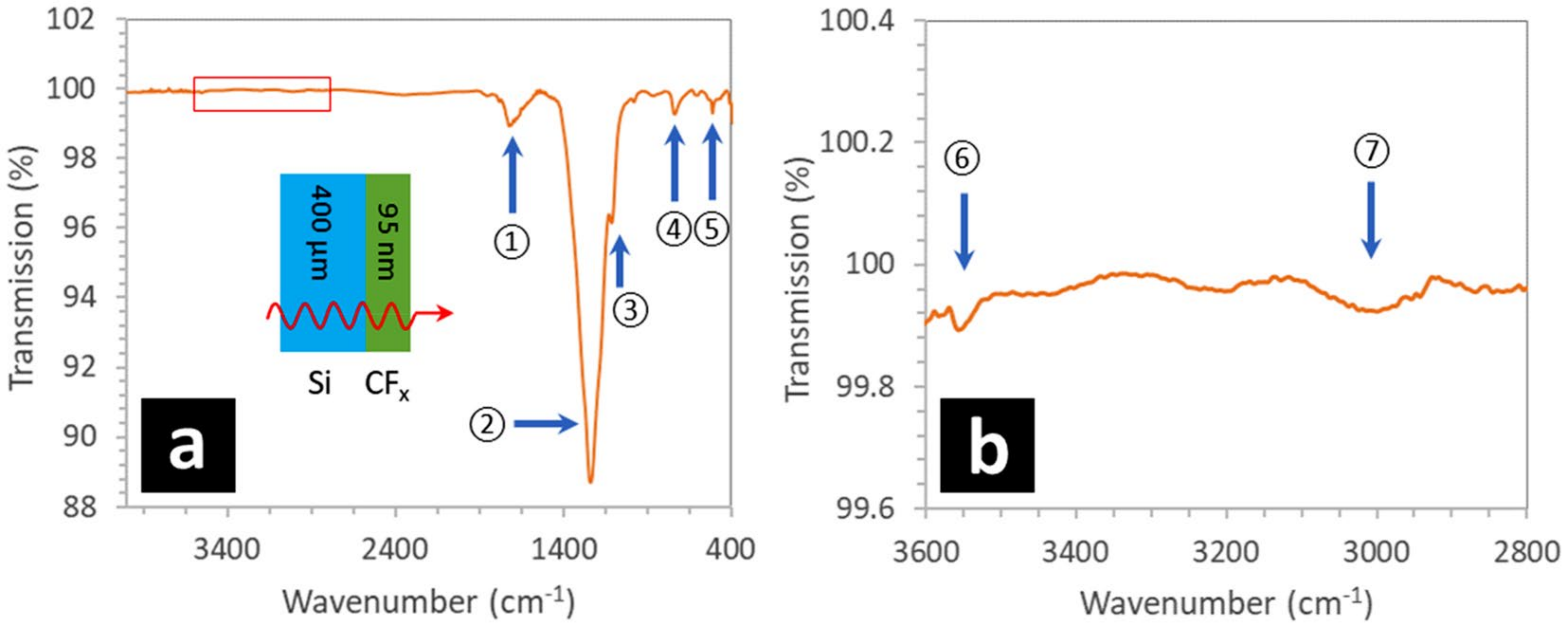
Fourier transform infrared spectroscopy (FTIR) of the 95 nm-thick fluoropolymer film deposited onto a lowly-doped p-type silicon wafer—see Methods. (**a**) The FTIR sweep from 400 to 4000 cm^−1^. (**b**) Zoom of the FTIR from 2700 to 3300 cm^−1^. The inset to (**a**) shows the setup with the fluoropolymer indicated as CF_x_. The fluoropolymer film is deposited onto the silicon wafer by PECVD using a CHF_3_ plasma. The red rectangle in (**a**) is the zoomed zone of the spectrum shown in (**b**). The correspondence of numbered peaks and their explanation can be found in the text.

Fluoropolymer films were analysed using Fourier transform infrared spectroscopy (FTIR)—see Methods. To do so, 95 nm-thick fluoropolymer films were prepared using PECVD (see Methods) on lowly-doped p-type silicon wafers—see Methods. Figure 14 shows the FTIR spectrum obtained when scanning from 400 to 4000 cm^−1^. Several observations can be made from this result. First of all, the FTIR spectrum corresponds very well with FTIR spectra of fluoropolymers deposited under similar conditions^30,70^. If we consider Fig. 14a, the peak around 1711 cm^−1^—peak ① in Fig. 14a—corresponds to unsaturated stretching modes^70^—which generally indicates C=C and/or C=O—and is often seen in PECVD fluoropolymer films^30^. The prominent peak around 1240 cm^−1^—peak ② in Fig. 14a—corresponds to a CF_x_ (x = 1–3)^70^—and symmetric and asymmetric CF_2_ stretches by Winder and Gleason^30^. The convoluted peak around 1113 cm^−1^—peak ③ in Fig. 14a—possibly corresponds to symmetrical CF_2_ stretching^71^. The small peak around 738 cm^−1^—peak ④ in Fig. 14a—is assigned as being the amorphous phase^72^. The other small peak around 513 cm^−1^—peak ⑤ in Fig. 14a—corresponds to CF_2_ ‘rock’^73^. Figure 14b shows a zoom of the FTIR spectrum of the fluoropolymer film between 2800 and 3600 cm^−1^. The peak around 3500 cm^−1^—peak ⑥ in Fig. 14b—corresponds to OH stretching due to surface humidity^30^. The absence of strong peaks around 2900 cm^−1^ ⑦ in Fig. 14b indicates there is very little hydrogen, incorporated as C-H, in the fluoropolymer film. The FTIR results suggest that our film corresponds chemically to fluoropolymer films deposited by PECVD using CHF_3_ under similar conditions^30,70^. Comparing with FTIR spectra of poly(tetrafluoroethylene) [–F_2_C–CF_2_–]_n_, major peaks occur at 1240 cm^−1^, 1150 cm^−1^, and 516 cm^−1^^74^.

### Modelling the CV curves and estimating the charge in the fluoropolymer

The high frequency capacitance of the semiconductor $C_{s}$ in a p-type and n-type MIS capacitor can be approximated using the following formulae^50^:

1 $$C_{s} = Sgn\left( {\psi_{s} } \right)\frac{{C_{FBS} }}{\sqrt 2 }\frac{{1 - e^{{ - {\raise0.7ex\hbox{${q\psi_{s} }$} \!\mathord{\left/ {\vphantom {{q\psi_{s} } {kT}}}\right.\kern-\nulldelimiterspace} \!\lower0.7ex\hbox{${kT}$}}}} }}{{\sqrt {e^{{ - {\raise0.7ex\hbox{${q\psi_{s} }$} \!\mathord{\left/ {\vphantom {{q\psi_{s} } {kT}}}\right.\kern-\nulldelimiterspace} \!\lower0.7ex\hbox{${kT}$}}}} + \left( {\frac{{q\psi_{s} }}{kT} - 1} \right)} }}$$

2 $$C_{s} = Sgn\left( {\psi_{s} } \right)\frac{{C_{FBS} }}{\sqrt 2 }\frac{{e^{{{\raise0.7ex\hbox{${q\psi_{s} }$} \!\mathord{\left/ {\vphantom {{q\psi_{s} } {kT}}}\right.\kern-\nulldelimiterspace} \!\lower0.7ex\hbox{${kT}$}}}} - 1}}{{\sqrt {e^{{{\raise0.7ex\hbox{${q\psi_{s} }$} \!\mathord{\left/ {\vphantom {{q\psi_{s} } {kT}}}\right.\kern-\nulldelimiterspace} \!\lower0.7ex\hbox{${kT}$}}}} - \left( {\frac{{q\psi_{s} }}{kT} + 1} \right)} }}$$

where $\psi_{s}$ is the semiconductor surface potential, $k$ is the Boltzmann constant, $q$ is the elementary charge, $T$ is the absolute temperatre, and $C_{FBS}$ is the flat-band capacitance ($\psi_{s}$ = 0) in the semiconductor which is given by:

3 $$C_{FBS} = \frac{{\varepsilon_{s} \varepsilon_{0} }}{\lambda }$$

where $\varepsilon_{s}$ is the dielectric constant of the semiconductor, $\varepsilon_{0}$ is the vacuum permittivity, and $\lambda$ is the extrinsic Debye length (doping density = $N$) in the semiconductor:

4 $$\lambda = \sqrt {\frac{{\varepsilon_{s} \varepsilon_{0} kT}}{{q^{2} N}}}$$

The insulator capacitance $C_{i}$ is given by:

5 $$C_{i} = \frac{{\varepsilon_{i} \varepsilon_{0} }}{{d_{i} }}$$

where $\varepsilon_{i}$ is the dielectric constant of the insulator and $d_{i}$ is the thickness of the insulator.

The minimum capacitance of an MIS capacitor is given by:

6 $$C_{min} = \frac{{\varepsilon_{i} \varepsilon_{0} }}{{d_{i} + \left( {\frac{{\varepsilon_{i} }}{{\varepsilon_{s} }}} \right)w_{m} }}$$

where $w_{m}$ is the maximum depletion width of the surface depletion layer in the semiconductor which is given by:

7 $$w_{m} = \sqrt {\frac{{4kT\ln \left( {N/n_{i} } \right)}}{{q^{2} N}}}$$

The gate voltage $V$ of the MIS capacitor is given by:

8 $$V = V_{i} + \psi_{s} + V_{FB}$$

where $V_{i}$ is the voltage across the insulator layer and $V_{FB}$ is the flat band voltage, i.e. the gate voltage required to achieve flat band in the semiconductor. The total capacitance $C$ of the MIS capacitor is given by:

9 $$C = \frac{{C_{i} C_{s} }}{{C_{i} + C_{s} }}$$

**Figure 15:**
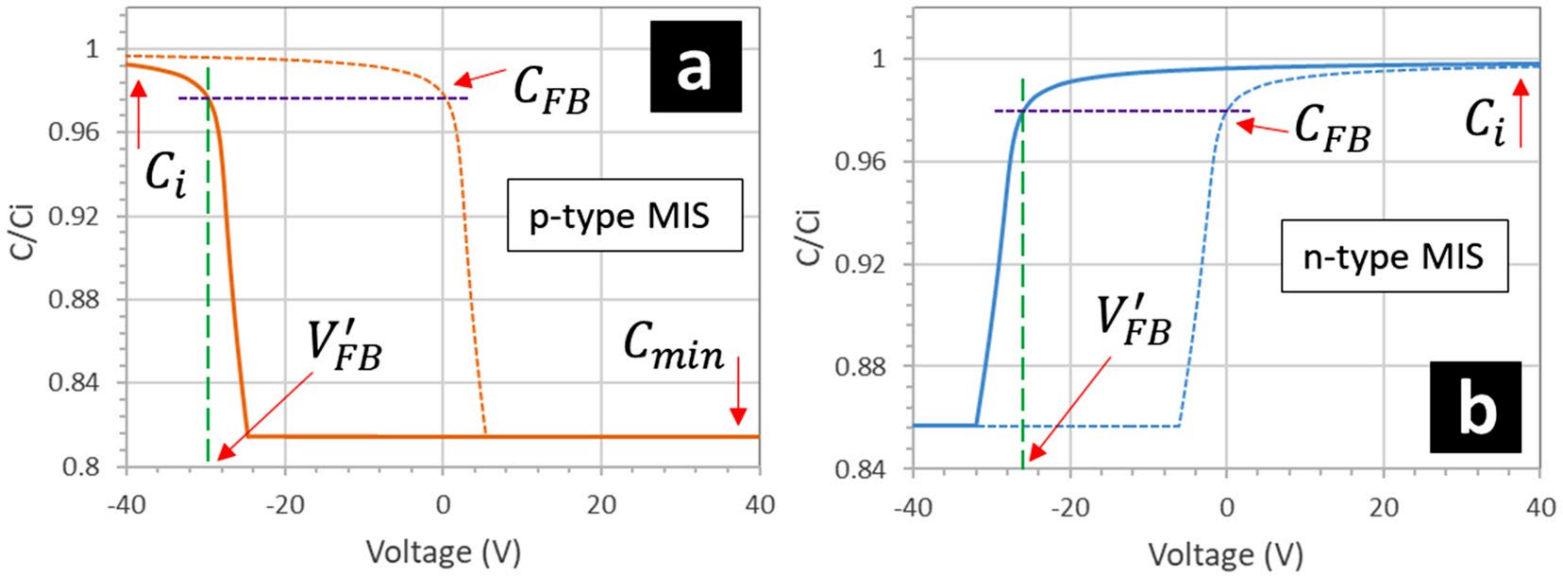
Modelled CV curves for (**a**) a p-type MIS capacitor and (**b**) an n-type MIS capacitor. $C_{i}$ is the insulator capacitance, $C_{min}$ is the minimum capacitance, $C_{FB}$ is the flat band capacitance, and $V_{FB}^{^{\prime}}$ is the shifted flat band voltage due to the presence of positive charges in the insulator.

These equations enable the capacitance of the MIS capacitor to be plotted as a function of applied voltage. Figure 15 shows the modelled CV curves for the p-type MIS (Fig. 15a) and the n-type MIS (Fig. 15b). The model uses the measured carrier concentrations and the experimentally-obtained value of $\varepsilon_{i}$. In addition to this, we know from above that the (positive) charge contained in the fluoropolymer has the effect of shifting the CV curves (p-type and n-type MIS) to the left. Thus, the voltage shift can be incorporated into the model. Figure 15 shows the CV curve of the p-type MIS and n-type MIS capacitor when a voltage shift equal to − 30 V (p-type) to − 26 V (n-type) is used. Clearly this modelled CV resembles what is observed experimentally on the + V to − V sweeps. The modelled normalized CV curved also predict a change in the capacitance equal to ~ 0.81 (p-type) and ~ 0.86 (n-type) due to carrier depletion/accumulation. The experimental results approximate these predicted changes: 0.86 ± 0.01 (p-type) and 0.93 ± 0.01 (n-type)—the observed change being larger in the p-type than the n-type. The reason for the discrepancy between the modelled curves and the experimental observations for the n-type silicon is not clear; although Fig. 13 indicates that the minimum capacitance is not experimentally reached—even at high negative bias.

The basic semiconductor equations for a p-type and n-type MIS capacitor^50^ can be rearranged to estimate the charge $Q_{i}$ present in the insulator:

10 $$Q_{i} = \frac{{\varepsilon_{i} \varepsilon_{0} }}{{d_{i} }}\left( {\phi_{m} - \left( {\chi + \frac{{E_{g} }}{2} + \frac{kT}{q}\ln \frac{{N_{A} }}{{n_{i} }}} \right) - V_{FB}^{^{\prime}} } \right)$$

11 $$Q_{i} = \frac{{\varepsilon_{i} \varepsilon_{0} }}{{d_{i} }}\left( {\phi_{m} - \left( {\chi + \frac{{E_{g} }}{2} - \frac{kT}{q}\ln \frac{{N_{D} }}{{n_{i} }}} \right) - V_{FB}^{^{\prime}} } \right)$$

where $\phi_{m}$ is the metal work function, $\chi$ is the semiconductor electron affinity, $E_{g}$ is the band gap of the semiconductor, $n_{i}$ is the intrinsic carrier concentration in the semiconductor, and $V_{FB}^{^{\prime}}$ is the shifted flat-band voltage of the CV curve. Using the calculated value of the flat-band capacitance $C_{FB}$ of the MIS:

12 $$C_{FB} = \frac{{C_{i} C_{FBS} }}{{C_{i} + C_{FBS} }}$$

the value of $V_{FB}^{^{\prime}}$ can be determined from the experimental CV curves of the p-type and n-type MIS capacitors. We are now in a position to use the measured values of $\varepsilon_{i}$, $d_{i}$, $N$, and $V_{FB}^{^{\prime}}$, together with the properties of the gold ($\phi_{m}$ = 5.1 eV) and the silicon ($E_{g}$ = 1.12 eV, $\chi$ = 4.05 eV, $n_{i}$ = 1 × 10^10^ cm^−3^) at a temperature of 300 K, and the fundamental constants ($\varepsilon_{0}$ = 8.85 × 10^12^ Fm^-1^, $k$ = 1.38 × 10^–23^ JK^-1^, $q$ = 1.6 × 10^–19^ C) to estimate the charge in the fluorocarbon insulator layer. Note that the energy gap of poly(tetrafluoroethylene) fluoropolymer has been measured to be ~ 7.7 eV^75^. Thin film fluoropolymer begins to strongly optically absorb around 150 nm or ~ 8 eV^76^. This is approximately the same as silicon dioxide used in MOS structures^49,50^. The election affinity of poly(tetrafluoroethylene) fluoropolymer is cited as ~ − 0.8 eV^77^, compared to 0.9 eV for silicon dioxide^78^. Using these values, the value of the positive charge in the fluoropolymer layer is calculated to be 5.72 × 10^–7^ C cm^−2^ (p-type MIS) and 5.11 × 10^–7^ C cm^−2^ -n-type MIS). By using a model for a metal-electret-semiconductor junction^79^, a similar value for the charge density can be obtained.

**Figure 16:**
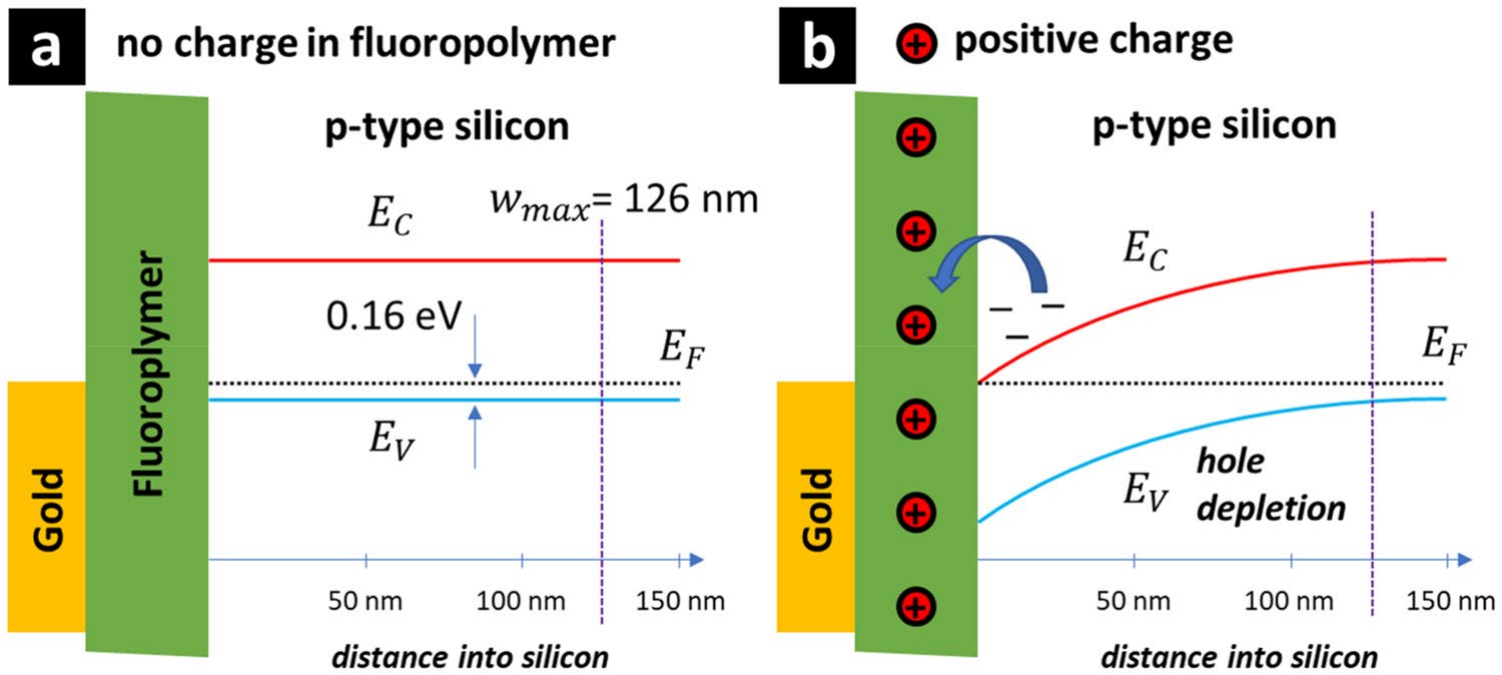
The effect of positive charge in the fluoropolymer on the energy band bending at the silicon surface of a moderately-doped p-type MIS capacitor. A gold/fluoropolymer/p-type silicon MIS capacitor (**a**) without and (**b**) with positive charge.

Figure 16 shows the effect of positive charge in the fluoropolymer on the energy band bending at the silicon surface of a moderately-doped p-type MIS capacitor. Note that the band-bending in Fig. 16 is not schematic but rather calculated by numerical differentiation of the Poisson equation. As above, the measured values, materials’ properties (gold, fluoropolymer, and silicon), and the fundamental constants are used for the calculation. In the absence of positive charge in the fluoropolymer, at zero applied voltage the MIS capacitor is in flat-band conditions—see Fig. 16a; this would imply carrier depletion at small voltages—something that is not observed here. In the case of positive charge in the fluoropolymer, band bending causes hole depletion and electron accumulation at the silicon surface. However, we know from the CV measurements that an inversion layer capacitance is not observed; this implies an electron trapping mechanism—indicated by the blue arrow in Fig. 16b. Given this information, we can now make some suggestions concerning the experimentally-observed losses in the fluoropolymer-coated CPW.

### Low surface losses and light insensitivity for fluoropolymer coated CPW

We are now able to suggest an explanation for the behaviour of the fluoropolymer-coated CPW at microwave frequencies.

**Figure 17:**
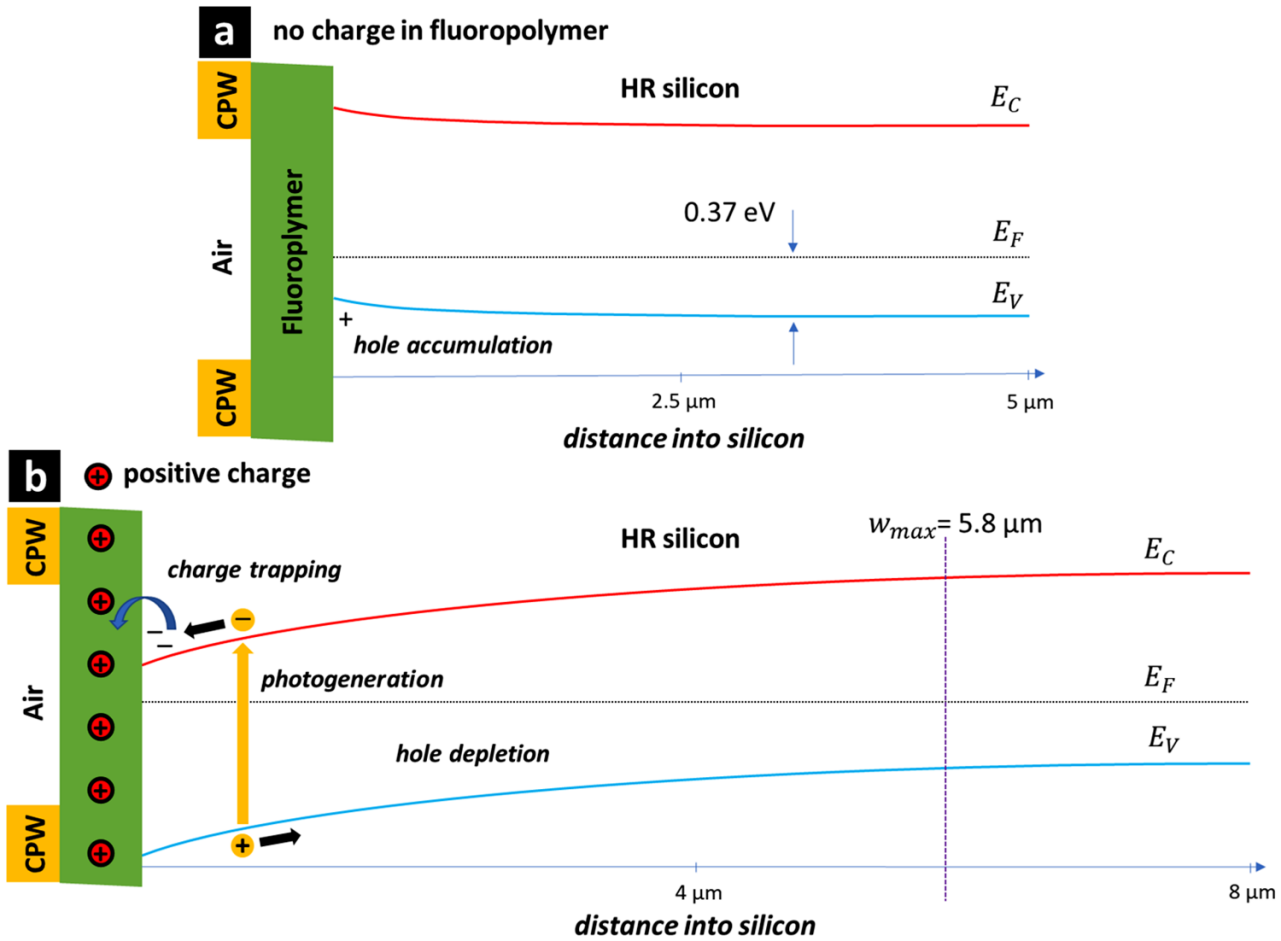
The effect of positive charge in the fluoropolymer on the energy band bending at the high-resistivity silicon surface positioned between the metal tracks of a CPW. The band diagram showing surface depletion, trapping, and optical generation of carriers—effect of the presence and lifetime of free carriers near the silicon surface between the CPW tracks. A fluoropolymer/high-resistivity silicon CPW intertrack surface (**a**) without and (**b**) with positive charge. The HR silicon doping is 1.46 × 10^13^ cm^−3^ (p-type).

Figure 17 shows the effect of positive charge in the fluoropolymer on the energy band bending at the HR silicon surface positioned between the metal tracks of a CPW. Again, the band-bending in Fig. 17 is not schematic but rather calculated by numerical differentiation of the Poisson equation. The doping in the HR silicon was assumed to be 1.46 × 10^13^ cm^−3^ (p-type). As above, the measured values, materials’ properties (gold, fluoropolymer, and silicon), and the fundamental constants are used for the calculation. Note however that the position of the CPW metal in Fig. 17 is purely schematic. In the case of hypothetic absence of positive charge in the fluoropolymer—Fig. 17a, band bending causes a hole accumulation at the silicon surface—such holes would contribute to intertrack losses. In addition, illumination would generate free carries which would contribute to losses in CPW—something that is not experimentally observed in the microwave measurements of the CPW. In the case of positive charge in the fluoropolymer—Fig. 17b, the energy band bending at the silicon surface causes hole depletion far from the silicon surface—these holes do not contribute to microwave losses in the CPW. In the presence of the charge the depletion width is ~ 6 µm, this is larger than the miniature CPW intertrack spacing where most of the microwave field energy is contained^26^. However, the band bending results in electron accumulation at the silicon interface. The experimental results (CPW and CV) indicate that these electrons are trapped at the surface or in the fluoropolymer; meaning that they also do not contribute to microwave losses in the CPW—this is also experimentally observed. In the case of illumination, photogenerated carriers near the surface will be accelerated in opposite directions by the electric field caused by the energy band bending. The holes will move to the right and not contribute to losses, the electrons to the left to be presumably trapped. This mechanism can be proposed as to why the fluoropolymer-coated CPW losses are highly insensitive to illumination. The experimental results suggest that the photogenerated electrons are relatively rapidly trapped (1/50 GHz = 20 ps) at or near to the fluoropolymer interface between the CPW tracks. However, more work is needed to clarify this last point.

### Advantages of plasma-deposited fluoropolymer in microwave circuitry

Along with the apparent benefits of being an electret demonstrated in this study, the fluoropolymer has other advantages with respect to RF circuity and its associated testing—this will be discussed here. First, fluoropolymer thin films are easy and relatively cheap to deposit as a back-end process—it is deposited without masking using plasma (PECVD) at room temperature. The fluoropolymer here seems to have a high breakdown field strength—this indicates a potential advantage for high bias electronics. Fluoropolymer is low loss up to very high frequencies—for example, polytetrafluoroethylene (PTFE) has a dielectric constant of ~ 2 and a loss tangent (tan $\delta$) < 5 × 10^–4^ from low frequency to 60 GHz. The benefits of a plasma-deposited thin film fluoropolymer in microwave electronics should be extendible to higher frequencies—100 GHz and above. Fluoropolymers have a broad operating temperature range (typically: − 200 °C to + 260 °C), they do not degrade in humidity or exposure to ultraviolet light, and they are both non-toxic and non-flammable. Thin film fluoropolymer is naturally hydrophobic—it renders a circuit water resistant/repellent and has a self-cleaning characteristic. Being impermeable to water, underlying silicon surfaces (and other surfaces) do not oxidize. Fluoropolymer is also chemically robust, meaning circuits and surfaces are protected from exposure to other chemicals. The fluoropolymer thin film can be removed in oxygen plasma if required. In terms of testing, the fluoropolymer does not adhere to commercial probes. The fluoropolymer is transparent to white light, enabling underlying circuitry to be visible when using an optical microscope. As relatively bright microscope light is often used in microwave measurement setups, suppression of effects due to photogenerated carriers in semiconductors such as silicon is thus an advantage. Finally, we have seen that the thin soft films of fluoropolymer are very easy to pierce using commercial microwave probes enabling reproducible, repeatable measurements.

## Conclusions

The signal losses associated with the free, non-passivated silicon surfaces between coplanar waveguide (CPW) metal tracks are sensitive to oxidation, humidity, temperature, and illumination. However, by depositing a low-loss, thin film (95 nm thick) of fluoropolymer coating, by plasma-enhanced chemical vapour deposition (PECVD) using trifluoromethane (CHF_3_), the miniature CPW intertrack surface-associated signal losses can be rendered insensitive to native oxidation, temperature, humidity, and illumination (~ 2400 lx) with white light. Electrical characterization of gold-fluoropolymer-silicon MIS capacitors indicates the presence of positive charge in the fluoropolymer. We conclude that the resulting band bending at the intertrack silicon surface, due to this positive charge, results in low losses due to the absence of free carriers at the silicon/fluoropolymer interface. The fluoropolymer film is deposited without masking at room temperature in one relatively rapid step (< 4 min). The transparency of the fluoropolymer to white light means that underlying circuitry is optically-visible and contact pads can be easily located using optical microscopy for manual or automatic characterization via probing. The mechanical properties of the fluoropolymer—together with its adequate surface adhesion—mean that common, commercial ground-signal-ground (GSG) microwave probes can easily locally pierce the fluoropolymer thin film and achieve excellent, repeatable electrical landing contact to CPW measurement pads. We believe that the simple maskless fluoropolymer passivation presented here lends itself well to the passivation of high-frequency silicon circuity and potentially microelectronics. Finally, and perhaps most interestingly from a scientific point-of-view, the study has revealed the importance of the link between a thin film, low-loss electret and semiconductor surface-associated losses in microwave microelectronics circuitry.

## Methods

### Design and Microfabrication of the CPWs

The CPW were designed using commercial electromagnetic modelling software (HFSS Ansys, USA). Commercial wafer-bonded^80^, Czochralski silicon-on-insulator (SOI) wafers (Si-Mat, Germany) were used to fabricate the CPW. The device layer (DL) thickness of the SOI is 20 µm, the buried silicon dioxide (BOX) thickness is 2 µm, and the silicon handle wafer thickness is 400 µm. The silicon crystal orientation is (100) and has an electrical resistivity of > 1000 ohms cm (p-type background, < 1.46 × 10^13^ cm^−3^)^81^. In terms of high-resistivity silicon, a resistivity of 1000 Ω cm falls outside the advised resistivity for low-loss RF systems (> 5000 Ω cm)^82^. It is thus challenging to render the surface-associated losses of lower-resistivity SOI material lower and stable in miniature CPW. All microfabrication was performed in a class ISO 5/7 cleanroom (*T* = 20 ± 0.5 °C; *RH* = 45 ± 2%)—all chemicals employed were VLSI-grade, except for solvents which were electronic-grade. A 50 nm-thick silicon dioxide layer was deposited onto the top of the silicon DL using low pressure chemical vapour deposition (LPCVD). The CPW metal tracks were then patterned onto the SOI wafer surface using electron beam lithographic masking (EBPG 5000 Plus—Raith, Germany), evaporation (MEB 550 S—Plassys, France), and lift-off techniques. The CPW lines were composed of chromium/gold (10/500 nm)—leading to contact pads having a thickness of 1 µm. A chromium/gold metallization was chosen due to its chemical resistance to buffered hydrofluoric acid^83^. One of the CPW samples has the miniature CPW portion of the line running across a silicon membrane. This membrane was created using front-to-back photolithographic masking techniques (MA/BA6 mask aligner—Suss Microtech, Germany) and by locally deep etching (PlasmaLab Pro 100 Estrelas—Oxford Instruments, UK) the 400 µm-thick silicon handle wafer underneath the miniature CPW. Following this, the BOX underneath the silicon DL membrane was removed using hydrofluoric acid. All deposited thin films (including verification of lithographic resists) were measured using a commercial precision surface profiler (DektakXT—Bruker, USA).

### Fabrication of the MIM and MIS capacitors

Commercial, single crystal silicon wafers (Siltronix, France) were used for the fabrication of the metal–insulator-metal (MIM) and metal–insulator-semiconductor (MIS). The silicon wafers were cleaned using a standard RCA method^84^. For the MIS capacitors, the rear surfaces of 3-inch diameter (100) boron-doped p-type (0.1–0.5 Ω cm—6.7 × 10^16^ cm^−3^ measured using CV) and (111) phosphorous-doped n-type (0.03–0.05 Ω cm—1.3 × 10^17^ cm^−3^ measured using CV) silicon wafers were implanted (depth ~ 50 nm) with boron and phosphorous to 1 × 10^20^ cm^−3^ respectively to give a very highly doped region^85^. The implanted dopants were activated using rapid thermal annealing (RTA) at 850 °C for 1 min in argon (AnnealSys, France). Following this, a 200 nm-thick aluminium thin film was evaporated onto the rear surface of the silicon wafer—and annealed at 450 °C for 30 s under forming gas (N_2_/H_2_) using RTA in a Jipelec-JetFirst (Semco Technologies, France)^86^. For the MIM capacitors, a 200 nm thick gold film was evaporated onto the surface of a 3-inch diameter (100) n-type silicon wafer (0.019–0.024 Ω cm to 1.1 × 10^18^ cm^−3^) to act as the bottom electrode. The 95 nm-thick fluoropolymer film was then deposited onto all wafers using PECVD (see below). To complete the MIM and MIS structures, patterned gold (200 nm) films were evaporated onto the fluoropolymer surface in a commercial electron beam evaporator (as above) and the fluoropolymer was deposited using PECVD as described above. For the MIS samples, prior to fluoropolymer disposition, the silicon surfaces were exposed to VLSI-grade (7:1 40%NH_4_F: /50%HF) buffered hydrofluoric acid (*p*H ~ 5) for 30 s. Note that care was taken to avoid damage to the aluminium-based ohmic contacts on the rear surfaces of the wafers by containing the buffered HF to the silicon top surface. The 200 nm-thick gold dots for the top electrical contacts of the MIM and the MIS were formed by evaporation gold via a physical ‘shadow mask’ composed of a thin film metal foil pierced with holes having nominal dimensions of 270 × 270 µm. Metals are known to have a poor adherence to fluoropolymers due to low surface energy^87,88^, shadow masking was therefore used for the metallization of gold/fluoropolymer/semiconductor junctions^89^ as this eliminates the need for post-processing, e.g. lift-off or lithographic patterning, which could jeopardize the adhesion of the gold contacts.

### Fluoropolymer deposition

The thin film fluoropolymer was deposited onto the silicon and SOI wafers using plasma enhanced chemical vapour deposition (PECVD) in a Plasmalab 80 + (Oxford Instruments, UK). A trifluoromethane (CHF_3_) plasma was used with the following parameters: CHF_3_ flow rate = 50 sccm, CHF_3_ pressure = 150 mTorr, Power = 180 W. The DC bias was recorded to be − 279 V. The thickness of the resulting fluoropolymer layer was measured to be 95 ± 2.2 nm, using a commercial precision surface profiler DektakXT (Bruker, USA), i.e. a deposition rate of ~ 25.3 ± 0.6 nm min^-1^ under these conditions—comparable with previous work^31,42,51,90,91^.

### Current–voltage, capacitance–voltage, and microwave measurements

The current–voltage (IV) measurements at DC were conducted using a probe station and a dual channel system SourceMeter (2612B—Keithley, USA). The capacitance–voltage (CV) measurements were conducted using a probe station and a calibrated Precision Impedance Meter (4294A—Agilent, USA). The small signal voltage was ± 0.1 V for all measurements. The maximum voltage step was 0.35 V (± 40 V bias). The delay time was 250 ms. The instrument plus probes was calibrated from 40 Hz to 110 MHz. The microwave measurements up to 50 GHz were conducted using a N5245A vector network analyser (VNA) (Agilent Technologies, USA) and commercial ground-signal-ground RF probes (Cascade Microtech, USA) having a pitch of 250 µm. Using a commercial impedance standard substrate, a line-reflect-reflect-match calibration of the VNA plus the RF probes was performed before the measurements. The input source power and the intermediate frequency bandwidth are set respectively to − 10 dBm (0.1 mW) and 100 Hz. The insertion loss *IL* of a two-port network is obtained from the forward s-parameter transmission coefficient: $IL = - 20\log_{10} \left| {s_{21} } \right|$. Note that the *IL* of the miniature CPW are not de-embedded from the whole system as the their losses dominate the total losses^26^. For sample illumination with bright white light, the irradiance of the microscope (Zoom z10-5 W LED—MPI Corp., USA) was measured using a large-band power meter (13PEM001—Melles Griot, Japan). During the time between the experiments, all samples were stored in wafers boxes in a controlled cleanroom environment.

### FTIR samples and measurements

The Fourier transform infrared spectroscopy of the fluoropolymer films was conducted in a commercial FT-IT spectrometer (Spectrum-2000—Perkin Elmer, USA). The samples were prepared using 2-inch diameter commercial (100) boron-doped p-type (5–10 Ω cm to ~ 2 × 10^15^ cm^-3^) silicon wafers. Following wafer cleaning and a subsequent surface treatment using VLSI-grade 7:1 buffered hydrofluoric acid for 30 s, a 95 nm-thick fluoropolymer film was immediately deposited on the silicon wafer surface using PECVD of CHF_3_—as described above.

## Supplementary Information

Supplementary Information.

## References

[1] Wen CP (1969). Coplanar waveguide: A surface strip transmission line suitable for nonreciprocal gyromagnetic device applications. IEEE Trans. Microw. Theory Tech..

[2] Collin RE (2001). Foundations for microwave engineering.

[3] Simons RN (2001). Coplanar waveguide circuits, components, and systems.

[4] Pozar DM (2012). Microwave engineering.

[5] Rappaport TS (2019). Wireless communications and applications above 100 GHz: Opportunities and challenges for 6G and beyond. IEEE Access.

[6] Saad W, Bennis M, Chen M (2020). A vision of 6G wireless systems: Applications, trends, technologies, and open research problems. IEEE Netw..

[7] Marzouk J (2015). MEMS probes for on-wafer RF microwave characterization of future microelectronics: design, fabrication and characterization. J. Micromech. Microeng..

[8] Taleb A, Sergiyenko O, Flores-Fuentes W, Mercorelli P (2020). Control and automation for miniaturized microwave GSG nanoprobing. Machine vision and navigation 751–768.

[9] Kandala A (2017). Hardware-efficient variational quantum eigensolver for small molecules and quantum magnets. Nature.

[10] Göppl M (2008). Coplanar waveguide resonators for circuit quantum electrodynamics. J. Appl. Phys..

[11] Mehrotra P, Chatterjee B, Sen S (2019). EM-wave biosensors: A review of RF, microwave, mm-wave and optical sensing. Sensors.

[12] Reyes AC (1995). Coplanar waveguides and microwave inductors on silicon substrates. IEEE Trans. Microw. Theory Tech..

[13] Gamble HS (1999). Low-loss CPW lines on surface stabilized high-resistivity silicon. IEEE Microw. Guid. Wave Lett..

[14] Schollhorn C, Zhao W, Morschbach M, Kasper E (2003). Attenuation mechanisms of aluminum millimeter-wave coplanar waveguides on silicon. IEEE Trans. Electron Devices.

[15] Pfeifer T, Heiliger H-M, Stein von Kamienski E, Roskos HG, Kurz H (1995). Charge accumulation effects and microwave absorption of coplanar waveguides fabricated on high–resistivity Si with SiO_2_ insulation layer. Appl. Phys. Lett..

[16] Lederer D, Raskin J-P (2003). Substrate loss mechanisms for microstrip and CPW transmission lines on lossy silicon wafers. Solid-State Electron..

[17] Rong B, Burghartz JN, Nanver LK, Rejaei B, vanderZwan M (2004). Surface-Passivated High-Resistivity Silicon Substrates for RFICs. IEEE Electron Device Lett..

[18] Lederer D, Raskin J-P (2005). Effective resistivity of fully-processed SOI substrates. Solid-State Electron..

[19] Neve, C. R. *et al.* Impact of Si substrate resistivity on the non-linear behaviour of RF CPW transmission lines. In *2008 European Microwave Integrated Circuit Conference* 36–39 (IEEE, 2008). 10.1109/EMICC.2008.4772222.

[20] Yuhang Z, Jiarong T, Xuan Z, Yong W (2009). A low-loss V-groove coplanar waveguide on an SOI substrate. J. Semicond..

[21] Chen C-J, Wang R-L, Su Y-K, Hsueh T-J (2011). A nanocrystalline silicon surface-passivation layer on an HR-Si substrate for RFICs. IEEE Electron Device Lett..

[22] Abuelgasim A (2011). Reduced microwave attenuation in coplanar waveguides using deep level impurity compensated Czochralski-silicon substrates. Semicond. Sci. Technol..

[23] Evseev SB, Nanver LK, Milosaviljevic S (2012). Surface-charge-layer sheet-resistance measurements for evaluating interface RF losses on high-resistivity-silicon substrates. IEEE Trans. Microw. Theory Tech..

[24] Bruno A (2015). Reducing intrinsic loss in superconducting resonators by surface treatment and deep etching of silicon substrates. Appl. Phys. Lett..

[25] Woods W (2019). Determining interface dielectric losses in superconducting coplanar-waveguide resonators. Phys. Rev. Appl..

[26] Marzouk J, Avramovic V, Arscott S (2020). Intertrack surface losses in miniature coplanar waveguide on silicon-on-insulator. J. Phys. Appl. Phys..

[27] Raveendran A, Sebastian MT, Raman S (2019). Applications of microwave materials: A review. J. Electron. Mater..

[28] Kressmann R, Sessler GM, Gunther P (1996). Space-charge electrets. IEEE Trans. Dielectr. Electr. Insul..

[29] Oehrlein GS, Zhang Y, Vender D, Haverlag M (1994). Fluorocarbon high-density plasmas: I—Fluorocarbon film deposition and etching using CF 4 and CHF 3. J. Vac. Sci. Technol. Vac. Surf. Films.

[30] Winder EJ, Gleason KK (2000). Growth and characterization of fluorocarbon thin films grown from trifluoromethane (CHF3) using pulsed-plasma enhanced CVD. Appl. Polym. Sci..

[31] Easwarakhanthan T, Beyssen D, Le Brizoual L, Bougdira J (2006). Spectroellipsometric analysis of CHF3 plasma-polymerized fluorocarbon films. J. Vac. Sci. Technol. Vac. Surf. Films.

[32] Chabal YJ, Higashi GS, Raghavachari K, Burrows VA (1989). Infrared spectroscopy of Si(111) and Si(100) surfaces after HF treatment: Hydrogen termination and surface morphology. J. Vac. Sci. Technol. A.

[33] Higashi GS, Chabal YJ, Trucks GW, Raghavachari K (1990). Ideal hydrogen termination of the Si (111) surface. Appl. Phys. Lett..

[34] Grant NE, Murphy JD (2017). temporary surface passivation for characterisation of bulk defects in silicon: A review. Phys. Status Solidi RRL Rapid Res. Lett..

[35] Raider SI (1975). Oxide growth on etched silicon in air at room temperature. J. Electrochem. Soc..

[36] Morita M, Ohmi T, Hasegawa E, Kawakami M, Ohwada M (1990). Growth of native oxide on a silicon surface. J. Appl. Phys..

[37] Gräf D, Grundner M, Schulz R, Mühlhoff L (1990). Oxidation of HF-treated Si wafer surfaces in air. J. Appl. Phys..

[38] Yablonovitch E, Allara DL, Chang CC, Gmitter T, Bright TB (1986). Unusually low surface-recombination velocity on silicon and germanium surfaces. Phys. Rev. Lett..

[39] Dubey G, Lopinski GP, Rosei F (2007). Influence of physisorbed water on the conductivity of hydrogen terminated silicon-on-insulator surfaces. Appl. Phys. Lett..

[40] Kalkofen B, Burte EP (2006). Sheet resistance increase of shallow doped silicon during native oxidation in air. ECS Trans..

[41] Morita M, Ohmi T, Hasegawa E, Kawakami M, Suma K (1989). Control factor of native oxide growth on silicon in air or in ultrapure water. Appl. Phys. Lett..

[42] Jansen HV, Gardeniers JGE, Elders J, Tilmans HAC, Elwenspoek M (1994). Applications of fluorocarbon polymers in micromechanics and micromachining. Sens. Actuators Phys..

[43] Oehrlein GS, Zhang Y, Vender D, Joubert O (1994). Fluorocarbon high-density plasmas: II—Silicon dioxide and silicon etching using CF 4 and CHF 3. J. Vac. Sci. Technol. Vac. Surf. Films.

[44] Endo K (1997). Fluorinated amorphous carbon as a low-dielectric-constant interlayer dielectric. MRS Bull..

[45] Jin Y-S, Kim G-J, Jeon S-G (2006). Terahertz dielectric properties of polymers. J. Korean Phys. Soc..

[46] Shkel YM, Klingenberg DJ (1996). Material parameters for electrostriction. J. Appl. Phys..

[47] Pelrine RE, Kornbluh RD, Joseph JP (1998). Electrostriction of polymer dielectrics with compliant electrodes as a means of actuation. Sens. Actuators Phys..

[48] Nicollian EH, Goetzberger A (1967). The Si-SiO _2_ interface: Electrical properties as determined by the metal-insulator-silicon conductance technique. Bell Syst. Tech. J..

[49] Sze SM (1981). Physics of semiconductor devices.

[50] Nicollian EH, Brews JR (1982). MOS (metal oxide semiconductor) physics and technology.

[51] Bariya AJ, Frank CW, McVittie JP (1990). A surface kinetic model for plasma polymerization with application to plasma etching. J. Electrochem. Soc..

[52] Fleetwood DM (1995). Border traps: Issues for MOS radiation response and long-term reliability. Microelectron. Reliab..

[53] Fleetwood, D. M. Fast and slow border traps in MOS devices. In *Proceedings of the Third European Conference on Radiation and its Effects on Components and Systems* 1–8 (IEEE, 1996). 10.1109/RADECS.1995.509743.

[54] Biswas N (2001). Electrical properties of fluorinated amorphous carbon films. J. Appl. Phys..

[55] Singh ThB (2004). Nonvolatile organic field-effect transistor memory element with a polymeric gate electret. Appl. Phys. Lett..

[56] Singh B, Marjanovic N, Sariciftci NS, Schwodiauer R, Bauer S (2006). Electrical characteristics of metal-insulator-semiconductor diodes and transistors with space charge electret insulators: towards nonvolatile organic memories. IEEE Trans. Dielectr. Electr. Insul..

[57] Yun M (2006). Capacitance-voltage characterization of polyfluorene-based metal-insulator-semiconductor diodes. Appl. Phys. Lett..

[58] Huang C, West JE, Katz HE (2007). Organic field-effect transistors and unipolar logic gates on charged electrets from spin-on organosilsesquioxane resins. Adv. Funct. Mater..

[59] Kalbitz R, Frübing P, Gerhard R, Taylor DM (2011). Stability of polarization in organic ferroelectric metal-insulator-semiconductor structures. Appl. Phys. Lett..

[60] Ismail, L. N. *et al.* Capacitance-voltage hysteresis of MIS device with PMMA:TiO_2_ nanocomposite as gate dielectric. In *RSM 2013 IEEE Regional Symposium on Micro and Nanoelectronics* 289–292 (IEEE, 2013). 10.1109/RSM.2013.6706532.

[61] Vais A (2017). On the distribution of oxide defect levels in Al_2_O_3_ and HfO_2_ high-k dielectrics deposited on InGaAs metal-oxide-semiconductor devices studied by capacitance-voltage hysteresis. J. Appl. Phys..

[62] Xia P (2017). Impact and origin of interface states in MOS capacitor with monolayer MoS2 and HfO2 High-k Dielectric. Sci. Rep..

[63] Lin J (2017). Examining the relationship between capacitance-voltage hysteresis and accumulation frequency dispersion in InGaAs metal-oxide-semiconductor structures based on the response to post-metal annealing. Microelectron. Eng..

[64] Fleetwood DM (2018). Border traps and bias-temperature instabilities in MOS devices. Microelectron. Reliab..

[65] Ren B (2018). Interface trap characterization of Al2O3/GaN vertical-type MOS capacitors on GaN substrate with surface treatments. J. Alloys Compd..

[66] Pazos SM, Aguirre FL, Tang K, McIntyre P, Palumbo F (2018). Lack of correlation between C-V hysteresis and capacitance frequency dispersion in accumulation of metal gate/high- *k* /n-InGaAs metal-oxide-semiconductor stacks. J. Appl. Phys..

[67] Sang L, Ren B, Liao M, Koide Y, Sumiya M (2018). Suppression in the electrical hysteresis by using CaF2 dielectric layer for p-GaN MIS capacitors. J. Appl. Phys..

[68] Ha T-J (2013). Transformation of the electrical characteristics of graphene field-effect transistors with fluoropolymer. ACS Appl. Mater. Interfaces.

[69] Yu S-Y, Wang K-H, Zan H-W, Soppera O (2017). Low-temperature sol–gel oxide TFT with a fluoropolymer dielectric to enhance the effective mobility at low operation voltage. Jpn. J. Appl. Phys..

[70] Capps NE, Mackie NM, Fisher ER (1998). Surface interactions of CF2 radicals during deposition of amorphous fluorocarbon films from CHF3 plasmas. J. Appl. Phys..

[71] Piwowarczyk J (2019). XPS and FTIR studies of polytetrafluoroethylene thin films obtained by physical methods. Polymers.

[72] Moynihan RE (1959). The molecular structure of perfluorocarbon polymers: Infrared studies on polytetrafluoroethylene. J. Am. Chem. Soc..

[73] Lau KKS, Caulfield JA, Gleason KK (2000). Structure and morphology of fluorocarbon films grown by hot filament chemical vapor deposition. Chem. Mater..

[74] Mark JE (2007). Physical properties of polymers handbook.

[75] Seki K (1990). Electronic structure of poly(tetrafluoroethylene) studied by UPS, VUV absorption, and band calculations. Phys. Scr..

[76] Yang MK, French RH, Tokarsky EW (2008). Optical properties of Teflon^®^ AF amorphous fluoropolymers. J. MicroNanolithography MEMS MOEMS.

[77] Zhang J, Darwish N, Coote ML, Ciampi S (2020). Static electrification of plastics under friction: The position of engineering-grade polyethylene terephthalate in the triboelectric series. Adv. Eng. Mater..

[78] Williams R (1965). Photoemission of Electrons from Silicon into Silicon Dioxide. Phys. Rev..

[79] Gunther P (1992). Determination of charge density and charge centroid location in electrets with semiconducting substrates. IEEE Trans. Electr. Insul..

[80] Lasky JB (1986). Wafer bonding for silicon-on-insulator technologies. Appl. Phys. Lett..

[81] Mallik, K., De Groot, C. H., Ashburn, P. & Wilshaw, P. R. Semi-insulating Czochralski-silicon for Radio Frequency Applications. In *2006 European Solid-State Device Research Conference* 435–438 (IEEE, 2006). 10.1109/ESSDER.2006.307731.

[82] Mallik K, de Groot CH, Ashburn P, Wilshaw PR (2006). Enhancement of resistivity of Czochralski silicon by deep level manganese doping. Appl. Phys. Lett..

[83] Williams KR, Gupta K, Wasilik M (2003). Etch rates for micromachining processing-Part II. J. Microelectromech. Syst..

[84] Kern W (1990). The evolution of silicon wafer cleaning technology. J. Electrochem. Soc..

[85] Pearson GL, Bardeen J (1949). Electrical properties of pure silicon and silicon alloys containing boron and phosphorus. Phys. Rev..

[86] Card HC (1976). Aluminum–silicon Schottky barriers and ohmic contacts in integrated circuits. IEEE Trans. Electron Devices.

[87] Siperko LM, Thomas RR (1989). Chemical and physical modification of fluoropolymer surfaces for adhesion enhancement: A review. J. Adhes. Sci. Technol..

[88] Sacher E (1994). Fluoropolymer metallization for microelectronic applications. Prog. Surf. Sci..

[89] Arscott S (2011). Moving liquids with light: Photoelectrowetting on semiconductors. Sci. Rep..

[90] Senkevich JJ, Tutor MJ, Sherrer DW (2000). Plasma-enhanced CVD of fluorocarbon thin films via CF3H/H2 chemistries. Chem. Vap. Depos..

[91] Yanev V (2004). Influence of the RF power on the deposition rate and the chemical surface composition of fluorocarbon films prepared in dry etching gas plasma. Surf. Sci..

