# Supplementary material for: Passivation of miniature microwave coplanar waveguides using a thin film fluoropolymer electret
Source: Sci Rep. 2021 Dec 16;11:24111. doi: 10.1038/s41598-021-03540-0 (PMC8677788; doi:10.1038/s41598-021-03540-0)
Supplement: Supplementary file 1 — Supplementary Information. [file 41598_2021_3540_MOESM1_ESM.docx]

*Supplementary Information for:*

**Passivation of miniature microwave coplanar waveguides using a thin film fluoropolymer electret**

Jaouad Marzouk^1^, Vanessa Avramovic^1^, David Guerin^1^ & Steve Arscott^1✉^

^1^University of Lille, CNRS, Centrale Lille, Univ. Polytechnique Hauts-de-France, UMR 8520-IEMN, F-59000 Lille, France. ✉email: [steve.arscott@univ-lille.fr](mailto:steve.arscott@univ-lille.fr)

**1. Supplementary results for CPW lines**


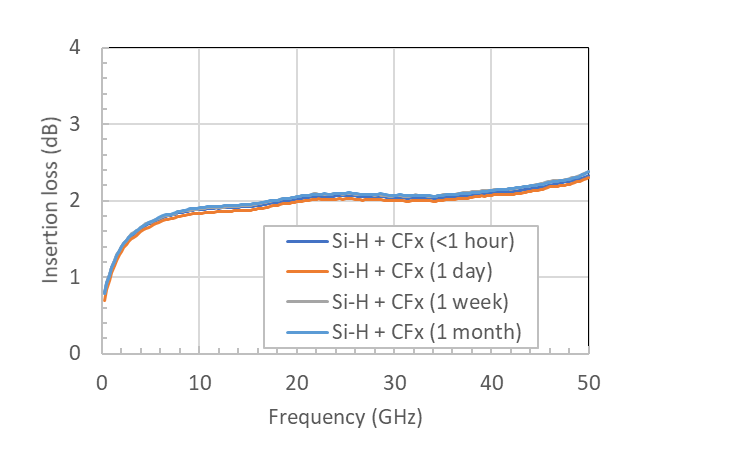


**Supplementary Figure 1.** Total insertion losses (dB) as a function of measurement frequency (GHz) for a large/small/large CPW circuit containing a miniature CPW portion (as in Figure 1 of the manuscript) having a length equal to 200 µm.


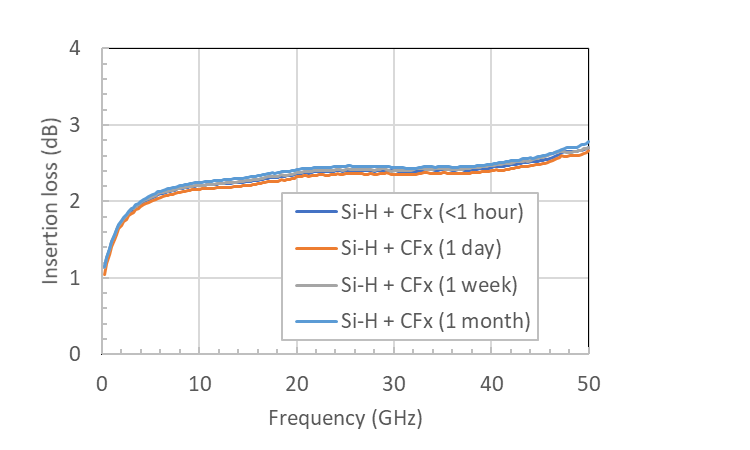


**Supplementary Figure 2.** Total insertion losses (dB) as a function of measurement frequency (GHz) for a large/small/large CPW circuit containing a miniature CPW portion (as in Figure 1 of the manuscript) having a length equal to 400 µm.


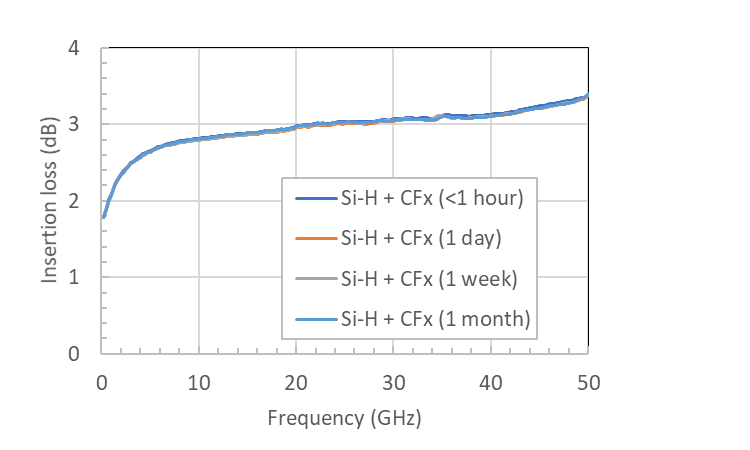


**Supplementary Figure 3.** Total insertion losses (dB) as a function of measurement frequency (GHz) for a large/small/large CPW circuit containing a miniature CPW portion (as in Figure 1 of the manuscript) having a length equal to 800 µm.


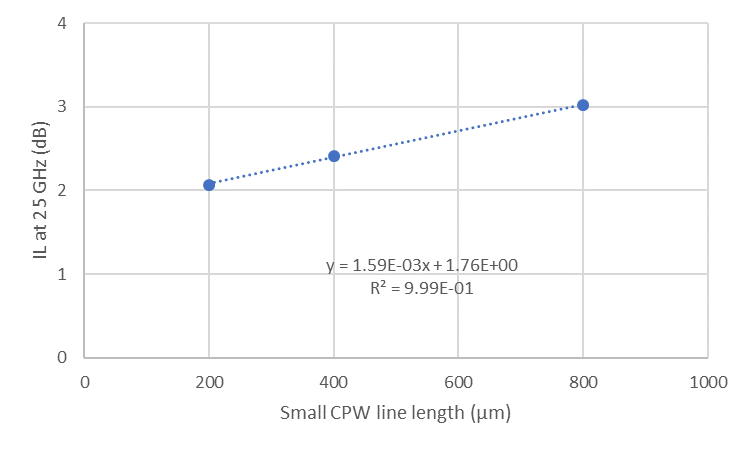


**Supplementary Figure 4.** The total insertion loss at 25 GHz plotted as a function of small CPW line length. The contribution of the large CPW portions of the system is ~1.76 dB


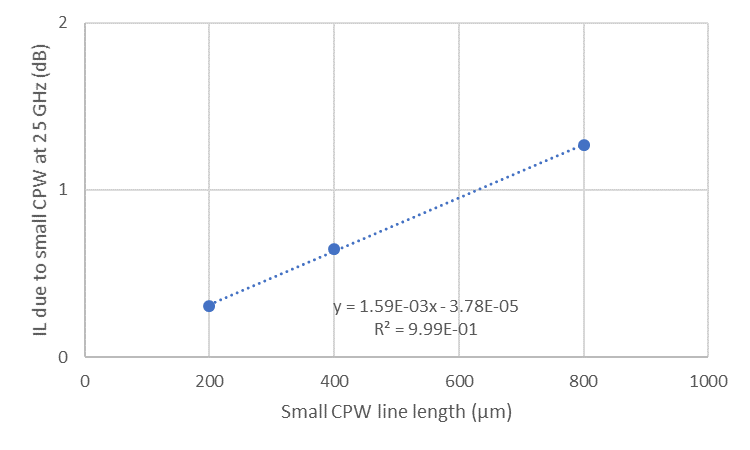


**Supplementary Figure 5.** The extracted insertion loss due only to the small CPW portions of the systems plotted as a function of small CPW line length.

These supplementary results enable the error of the microwave measurements to be evaluated at 0.03 dB. The insertion loss per distance of the small CPW lines is calculated to be 1.6 dB/mm when the lines are coated with the 95 nm thick fluoropolymer. This remains constant over 1 month with an error of 0.03 dB.

**2. Supplementary CV measurements**

Variations were observed in the absolute values of the CV (max and min) but the overall trends were repeated from device to device.


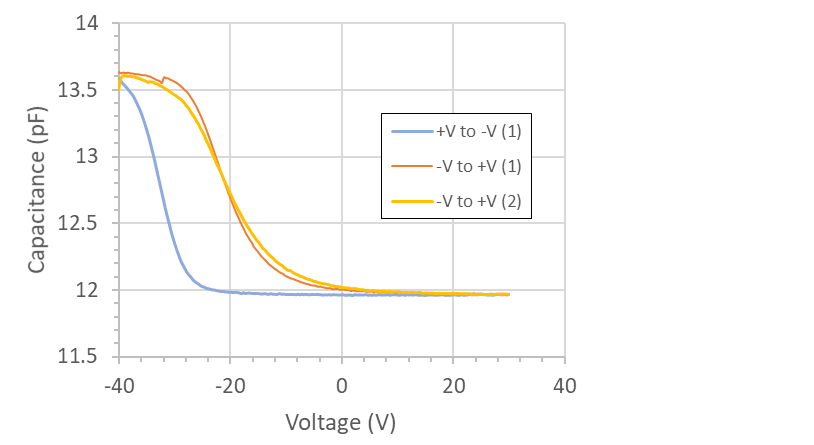


**Supplementary Figure 6.** Capacitance versus voltage for an p-type MIS at 100 KHz. Compare with Figure 12b of the manuscript.


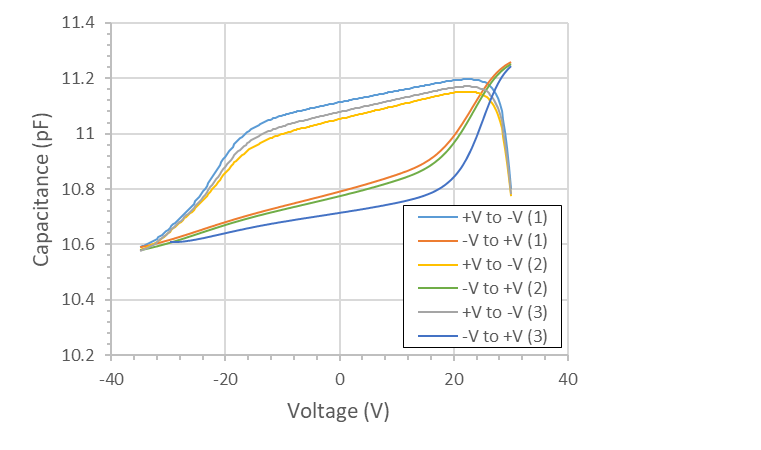


**Supplementary Figure 7.** Capacitance versus voltage for n-type MIS at 20 MHz. Compare with Figure 13d of the manuscript
